# Modulation of Cross-Language Activation During Bilingual Auditory Word Recognition: Effects of Language Experience but Not Competing Background Noise

**DOI:** 10.3389/fpsyg.2022.674157

**Published:** 2022-02-24

**Authors:** Melinda Fricke

**Affiliations:** Department of Linguistics, University of Pittsburgh, Pittsburgh, PA, United States

**Keywords:** individual differences in language processing, speech perception in noise, auditory word recognition, heritage speakers, bilingualism, cognate effects

## Abstract

Previous research has shown that as the level of background noise increases, auditory word recognition performance drops off more rapidly for bilinguals than monolinguals. This disproportionate bilingual deficit has often been attributed to a presumed increase in cross-language activation in noise, although no studies have specifically tested for such an increase. We propose two distinct mechanisms by which background noise could cause an increase in cross-language activation: a phonetically based account and an executive function-based account. We explore the evidence for the phonetically based account by comparing cognate facilitation effects for three groups of native English listeners (monolinguals, late (L2) learners of Spanish, and heritage Spanish speakers) and four noise conditions (no noise, speech-shaped noise, English two-talker babble, and Spanish two-talker babble) during an auditory lexical decision task in English. By examining word recognition in the dominant language, the role of language control mechanisms is minimized, and by examining three different types of competing noise, the role of energetic vs. informational masking can be assessed. Contrary to predictions, we find no evidence that background noise modulates cross-language activation; cognate facilitation is constant across the four noise conditions. Instead, several indices of word recognition performance are found to correlate with aspects of linguistic experience: (1) The magnitude of the cognate facilitation effect is correlated with heritage listeners’ self-ratings of Spanish proficiency; (2) Overall noise deficits are marginally larger for heritage listeners with lower English vocabulary scores; (3) Heritage listeners’ Spanish self-ratings predict their magnitude of informational masking; (4) For all bilinguals, the degree of masking incurred in both English and Spanish two-talker babble is correlated with self-reported daily exposure to Spanish; and (5) The degree of masking incurred by Spanish babble is correlated with Spanish vocabulary knowledge. The results enrich our understanding of auditory word recognition in heritage speakers in particular and provide evidence that informational masking is most subject to modulation due to variation in linguistic experience. It remains to be seen whether cross-language activation is modulated by noise when the target language is the less dominant one.

## Introduction

Non-native listeners are likely keenly aware that speech perception difficulties in adverse listening conditions appear amplified in a less-proficient language (see Garcia Lecumberri et al., 2010 and Scharenborg and van Os, 2019 for reviews). Indeed, findings have often indicated that second language listeners suffer disproportionately in noise as compared to their native language counterparts, especially in tasks involving whole-word perception (Black and Hast, 1962; Cooke et al., 2008; Morini and Newman, 2020). There are many reasons for this: non-native listeners are likely to have less robust phonetic (Hazan and Simpson, 2000; Cutler et al., 2004, 2008), phonological (Weber and Cutler, 2004; Broersma and Cutler, 2011), and lexical (Gollan et al., 2008; Gollan et al., 2011a; Shook et al., 2015) representations than natives, and as a result, they are more likely to experience bottlenecks in linguistic processing (Krizman et al., 2017), all of which may also adversely impact their ability to take advantage of contextual (Bradlow and Alexander, 2007; Skoe and Karayanidi, 2019) and semantic (Golestani et al., 2009) information so as to offset processing difficulties at other levels of representation.

Bilingual listeners also have to contend with cross-language activation from the non-target language. Decades of research on bilingual word recognition, both visual and auditory, have demonstrated that cross-language activation is pervasive in bilingual processing (e.g., Caramazza and Brones, 1979; Spivey and Marian, 1999; Van Hell and Dijkstra, 2002; Lagrou et al., 2011). Noisy listening conditions would only seem to exacerbate this issue, and indeed, many researchers have suggested exactly this (Rogers et al., 2006; Krizman et al., 2017; Morini and Newman, 2020). To our knowledge, however, no study has directly supported this hypothesis; other than speech perception in noise (“SPIN”) being more difficult in a non-native language, we are aware of no direct evidence that cross-language activation processes are altered by the presence of background noise.

This is the primary research question of the current study: *are* cross-language activation processes modulated by the presence of background noise, and if so, under what circumstances and for which listeners?

## Background

### Bilingual Speech Perception in Noise

Following recent calls to treat language experience-related predictors as continuous, rather than categorical, variables (Luk and Bialystok, 2013; Birdsong, 2018), we use the term “bilingual” broadly to refer to any individuals with knowledge of more than one language. We acknowledge, however, that this usage is distinct from much of the literature cited here.

The vast majority of studies examining bilingual SPIN has focused on perceptual deficits in a non-natively acquired language. Listeners who acquired the target language later in life are typically more strongly affected by adverse listening conditions as compared to listeners who acquired the language earlier, and this generalization holds for comparisons of natives vs. non-natives (Garcia Lecumberri and Cooke, 2006; Scharenborg et al., 2018) as well as earlier vs. later L2 learners (Mayo et al., 1997; Meador et al., 2000). Several studies have specifically implicated age of acquisition (“AoA”) as an explanatory variable (Mayo et al., 1997; MacKay et al., 2001), but given nearly unavoidable confounds between AoA and other potentially explanatory variables (dominance, proficiency, length of exposure, and context of acquisition), more work is still needed to understand their independent contributions, an issue to which we return below.

SPIN deficits also vary depending on the level of linguistic processing. Studies focusing on lower level phonetic perception, such as consonant and vowel identification, have most often found that the deficit for non-native as compared to native listeners remains constant even as the amount of noise increases (Takata and Nábělek, 1990; Hazan and Simpson, 2000; Cutler et al., 2004). Studies investigating higher level processing, on the other hand, have observed disproportionate deficits for non-natives. Tasks involving word recognition, whether in a sentence context (Mayo et al., 1997; Bradlow and Alexander, 2007) or not (Tabri et al., 2011; Scharenborg et al., 2018; Morini and Newman, 2020), have more often found that in increasing levels of noise, non-native performance drops off at a faster rate relative to natives. This suggests that bilingual *lexical* processing may be particularly disrupted in noise and/or that focusing on word recognition in noise may provide a window into a critical nexus of processing demands.

This latter point also intersects with an important distinction made in the literature on SPIN, that of energetic vs. informational masking (Cooke et al., 2008). Energetic masking refers to the idea that interfering sounds can render the target speech inaudible due to overlapping time and frequency characteristics of the sounds (e.g., being unable to hear a word because of a train rushing by), while informational masking refers to the decrement in recognition performance that occurs due to the informational content of non-target sounds (e.g., the ability of nearby speech to capture the listener’s attention). As such, informational masking is a catch-all term that encompasses a variety of cognitive-linguistic phenomena that do not necessarily form a unitary construct.

In an effort to understand the components of informational masking, studies have varied both the informational content of the masking sounds and the linguistic background of the listeners. Such work has demonstrated that listeners’ facility in deriving informational content from competing sounds plays a role in the degree of informational masking they experience. While it is known that a competing talker provides the equivalent of 6–8 dB *less* masking as compared to constant (“stationary”) noise at the same level (Festen and Plomp, 1990), the reason for this is that in the presence of competing speech, the target signal can still be “glimpsed” (Peters et al., 1998; Cooke, 2006), i.e., the time-frequency characteristics of speech are such that only bits and pieces of the target speech will be covered up by the competing speech at any moment in time. When the benefit afforded by glimpsing is taken into account, competing speech is generally *more* disruptive than non-speech noise, but also *variably* more disruptive. The reason is that cognitive factors, such as the ability to maintain attention on the target speech and suppress the activation of non-target representations, become a crucial determinant of the degree of informational masking experienced by the listener. Consequently, the maximum degree of informational masking has been reported to occur with two competing talkers (Freyman et al., 2004), because as the number of competing talkers increases, listeners’ capacity to derive informational content from the masker becomes swamped.

In the same vein, proficiency in the language of the masker also plays a role. Work by Van Engen and Bradlow (2007) and Van Engen (2010) demonstrated that for both native and non-native listeners, the ability to understand competing speech is associated with reduced word recognition accuracy. And while these studies compared listeners with either native or no proficiency in the masking language (see also Cooke, 2006), some studies have also found differential susceptibility to disruption from competing speech as a function of smaller gradations of proficiency in the masking language (Imai et al., 2005; Kilman et al., 2014).

Importantly, however, most studies examining the role of language experience in SPIN have involved group-level comparisons, sometimes with relatively little detail given about the participants themselves, and often relying on self-ratings of language ability rather than objective measures of language knowledge (but see Ezzatian et al., 2010; Van Engen, 2010; Warzybok et al., 2015; Scharenborg et al., 2018). As a result, it is as yet largely unknown which aspects of differential performance between listener groups should be attributed to differences in age of acquisition *per se* versus dominance, proficiency (and if so, which aspects), or domain-general cognitive abilities. In short, the *mechanisms* relating language experience to SPIN are not yet clear, and this will remain the case until the multitude of factors related to language experience can be disentangled.

### The Special Case of Heritage Listeners

In line with our broad usage of the term “bilingual,” we adopt the similarly broad definition of heritage bilingualism put forth by Rothman (2009), who proposes that a heritage language is “a language spoken at home or otherwise readily available to young children, and crucially this language is not a dominant language of the larger (national) society,” and a heritage speaker is someone who “has some command of the heritage language acquired naturalistically (e.g., Valdés, 1995, 2000).” This usage encompasses both simultaneous and sequential child bilingualism, and it acknowledges the fact that the distinction between these categories is in many cases blurred, depending on how many members of the family and immediate social network are fluent in the minority and/or majority languages. What heritage speakers of all profiles have in common is that they typically become more dominant in the societal majority language after beginning schooling. Heritage bilinguals’ language processing behavior is therefore likely to be similar in some ways to that of monolinguals (due to their early AoA and implicit acquisition) and in other ways to that of L2 late learners (due to their varying degrees of experience and ultimate attainment), but the details of processing behavior in this population remain poorly understood. Moreover, while these observations have been made in the literature in reference to knowledge of the heritage language itself (Montrul et al., 2008; Bolger and Zapata, 2011), especially in the context of SPIN, it bears some discussion that they could equally apply to the later-acquired majority language; we return to this point below.

In the United States, heritage speakers of Spanish generally acquire Spanish in the home from birth, and later become more dominant in English due to a relative lack of societal and community support for Spanish language use (Rothman, 2009). They may begin learning English at birth, simultaneously with Spanish, or slightly later, in young childhood, as their exposure to mainstream, English-dominant culture increases. Given the language context of the United States, the vast majority will ultimately participate in the English-speaking school system and workforce, and as a result, the quantity and quality of English exposure over the life span will be relatively comparable across individuals, with the main differences in English exposure relating to the nature of English input received before the onset of schooling around age four to five. The profile of Spanish experience across the life span, by contrast, may differ more dramatically depending on individual circumstances.

Investigating language processing behavior in US heritage speakers of Spanish therefore affords a unique opportunity to observe how the psycholinguistic processing of a dominant language can be modulated by linguistic experience in a non-dominant language, when differences in AoA for the two are minimized. This question is of great interest in the context of recent perspectives on bilingualism that place the role of linguistic experience and plasticity across the life span front and center for advancing our understanding how the cognitive system accommodates the presence of multiple languages (Baum and Titone, 2014; Kroll et al., 2014). From this standpoint, it is somewhat surprising that relatively little is known regarding language processing in heritage Spanish speakers. While some recent studies have investigated Spanish sentence processing in this population (Jegerski, 2018; Jegerski and Sekerina, 2020), and one study compared Spanish heritage speakers’ English sentence processing to that of English monolinguals (Bice and Kroll, 2021), most investigations of heritage speakers’ linguistic abilities have focused on offline knowledge of Spanish grammatical structures, using tasks, such as grammaticality judgments or structure elicitation tasks (e.g., Montrul, 2009; Montrul and Bowles, 2009). As a result, little is known about how factors such as dominance and proficiency influence basic online language processing in this population, in either Spanish or English.

One partial exception to this statement, however, is heritage Spanish speakers’ English auditory word recognition in adverse listening conditions. Perhaps surprisingly, given their lifetime of experience and consequent dominance in English, heritage Spanish listeners have repeatedly shown deficits in English SPIN relative to monolinguals. Two widely cited early studies found that despite acquiring English before age six and demonstrating monolingual-like performance (at ceiling) in the clear, heritage Spanish listeners’ English word recognition abilities in noise lagged behind that of their monolingual English counterparts. Mayo et al. (1997) found that heritage listeners required more favorable signal-to-noise ratios (SNRs) and derived less benefit from context as compared to monolinguals, and Rogers et al. (2006) found lower word shadowing accuracy at three SNRs and in two different adverse listening conditions. See also Tabri et al. (2011) for similar results in a somewhat comparable group of Arabic-English early bilinguals. In both of these studies, the patterns seemed to hold whether participants had acquired English simultaneously with Spanish since birth or had begun learning English slightly later, but both studies suffered from small sample sizes, with just 12 heritage bilinguals in each. Moreover, neither study arguably included any objective, independent measures of linguistic proficiency, making it difficult to identify the source of the observed deficits. Rogers et al. did report, though, that their heritage Spanish and monolingual English groups were matched on English accentedness, suggesting that English phonetic knowledge was at least somewhat comparable across groups.

More recently, two studies including more participants and more measures of linguistic performance have further explored heritage listeners’ English word recognition in noise. Krizman et al. (2017) tested 25 adolescent heritage bilinguals on a battery of perceptual tasks. They found that heritage bilinguals performed worse than monolinguals for sentence perception in noise, equivalently to monolinguals for single word perception in noise, and better than monolinguals for pure tone detection in noise. Morini and Newman (2020) tested 32 heritage bilinguals’ word recognition and word learning abilities in noise and found bilingual deficits (relative to monolinguals) only in the recognition task. Taken together, these studies support the idea that processes involved in retrieving linguistic representations from memory may be a significant source of difficulty for bilinguals charged with processing speech in noise, but they leave open the question of whether factors known to impact the retrieval process, such as the strength of cross-language activation and/or proficiency in the non-dominant language, may interact with the presence of noise.

Intriguingly, one small study found a relationship between L2 proficiency and SPIN performance in L1 *for late L2 learners*: von Hapsburg and Bahng (2009) reported that among native Korean late learners of English, greater L2 English proficiency was associated with worse L1 Korean word recognition in noise. This is particularly striking in the context of the plasticity-oriented perspectives highlighted above (Baum and Titone, 2014; Kroll et al., 2014). The process of acquiring and strengthening L2 representations and processing routines requires the learner to integrate new information into an already established system, a process that entails adaptation of the L1. As such, the development of proficiency in the L2 is ultimately a question of plasticity of the language processing system, i.e., the flexibility of the cognitive architecture that supports both the native language and any subsequently learned languages. While it is already widely accepted that the L1 should have a strong influence on the L2, perhaps especially in contexts of increased processing demands, such as adverse listening conditions, what this perspective underscores is that successful L2 acquisition may in some cases be associated with less optimal L1 performance.

To investigate these issues in more detail, the present study compares English word recognition in heritage Spanish bilinguals to that of both monolingual English and native English-late L2 Spanish bilinguals. Before introducing the study, however, we first provide an overview of what is known regarding cross-language activation in bilingual auditory word recognition.

### Cross-Language Activation in Bilingual Auditory Word Recognition

While the majority of evidence for cross-language activation in bilingual language processing comes from studies of visual word recognition (see Dijkstra and van Heuven, 2018, for a recent review), there is also considerable evidence that bilinguals experience cross-language activation in the auditory modality. Many studies in this vein have employed the visual world paradigm, often finding that bilingual listeners are more likely to look at an interlingual distractor picture (e.g., a duck—Spanish *pato*—when the target word is English “pot”) as compared to unrelated distractor pictures (Spivey and Marian, 1999; Marian and Spivey, 2003). Such findings indicate that representations in the non-target language are activated in the course of recognizing words in the target language. While cross-language activation appears robust when the non-target language is the dominant one (Blumenfeld and Marian, 2007; Chambers and Cooke, 2009), some studies have reported non-target activation of the non-dominant language as well, and it is likely that the presence of cross-language activation when the non-target language is *non-dominant* depends on factors such as AoA/proficiency (Canseco-Gonzalez et al., 2010) and the language immersion context (Spivey and Marian, 1999, whose participants had been immersed in the L2 for an average of 4 years).

There is some evidence for cross-language activation during auditory word recognition from non-visual world paradigms as well, but the data are actually rather sparse concerning auditory recognition of the type of “between language” words that share extensive cross-language overlap and that have often been employed in the literature on visual word recognition. The logic in these studies is that to the extent that representations in the non-target language become active in the course of target word recognition, words that overlap in form across languages should show differential processing as compared to control items. This prediction has been borne out in the auditory modality in several studies that have found differential recognition of interlingual homophones (Schulpen et al., 2003; Lagrou et al., 2011) and cognates (Woutersen et al., 1995; Blumenfeld and Marian, 2007; Guediche et al., 2020) as compared to control words, but on the whole, more data are needed in order to understand the relationships among cross-language overlap, language-specific phonetic cues, and cross-language activation patterns in the auditory modality.

#### Mechanisms by Which Cross-Language Activation Could Increase in Noise

In the auditory modality, language-specific phonetic cues could help bilinguals restrict activation to representations in the target language. Even words that share the same coarse-grained phonemic units across languages will generally be pronounced in such a way as to make the intended language clear, i.e., cognates and interlingual homophones will be realized with different “accents” depending on the language being spoken. The empirical record is quite mixed, however, with a handful of studies reporting that participants could take advantage of such language-specific phonetic cues (Schulpen et al., 2003; Ju and Luce, 2004; Fricke et al., 2016), and others finding they could not (Lagrou et al., 2011; McDonald and Kaushanskaya, 2020). Critically for the present study, even if some participants are capable of exploiting language-specific cues when listening conditions are favorable, the ability to do so should be greatly reduced in the presence of competing noise as a result of energetic and/or informational masking (Mattys et al., 2014). This should in turn lead to increased activation of words in the non-target language. We refer to this hypothesis as a *phonetically based account* of increased cross-language activation in the presence of background noise: to the extent that noise makes bilingual listeners unable to exploit phonetic cues to language membership, competing lexical representations in the non-target language should be more active in noise as compared to in the clear.

An alternative, though not mutually exclusive, possibility is that the necessity of directing cognitive resources toward the tasks of isolating and tracking the target speech stream could reduce the resources available for language control processes. Noisy listening conditions are understood to qualitatively alter the dynamics of lexical competition in the native language (McQueen and Huettig, 2012; Brouwer and Bradlow, 2016; Scharenborg et al., 2018) and recent perspectives on SPIN place the role of cognitive load front and center (see Peelle, 2018, and Pichora-Fuller et al., 2016 for reviews). During bilingual word recognition, inhibitory control in particular is known to play a role in resolving cross-language competition (Blumenfeld and Marian, 2013; Mercier et al., 2014; Chen et al., 2017), and it remains an open question to what extent other aspects of executive function may be involved as well (Kroll and Bialystok, 2013; Antoniou, 2019). An alternative to the phonetically based account is therefore an *executive function-based account*: to the extent that comprehending speech in noise taxes the cognitive system, fewer cognitive resources may be available for managing activation of the non-target language, resulting in greater cross-language activation in noise.

Importantly, the presence or absence of increased cross-language activation in noise will not enable us to distinguish between these two accounts. However, since language control processes are more likely to be engaged when listening in the less dominant language (i.e., when the non-target language is more dominant; Mercier et al., 2014; see also Green, 1998 and Misra et al., 2012), the present study provides a stronger test of the phonetically based account. By examining recognition of the dominant language by proficient speakers of a non-dominant language, the current study tests the effects of noise on cross-language activation while minimizing the role of language control processes.

### The Present Study

The present study investigates English auditory word recognition in three groups of listeners, all self-identified native English speakers, in the presence of three types of competing noise: speech-shaped noise, English two-talker babble, and Spanish two-talker babble. The inclusion of three distinct populations of native English listeners allows us to explore the questions of how proficiency and context of acquisition of the non-dominant language impact cross-language activation processes in noise, and the inclusion of three distinct types of noise allows us to examine the impact of the content of the noise itself.

The strength of cross-language activation was operationalized by measuring the extent of any cognate facilitation effects. We predicted that an increase in non-target language activation would boost recognition accuracy and speed for cognates relative to control words, such that any deleterious effects of noise on word recognition would be less severe for cognates relative to controls. Thus, while cognates should still be recognized with more difficulty in noise as compared to in the clear, the decrement in recognition performance may be less for cognates relative to control words.

The focus on native language processing in this study has several motivations. For one, the question of variation in native language processing as a function of language experience is compelling from a plasticity-oriented viewpoint. The present listeners all self-identify as native English speakers and consider English their more dominant language. The measures on which they differ most dramatically concern their experience with Spanish, allowing for an exploration of how aspects of this experience might impact processing in the more dominant language. The inclusion of two relatively large and qualitatively distinct bilingual participant groups also enables both group-based and individual differences-based analyses, helping to clarify whether differences across groups are easily captured by existing metrics (e.g., if the roles of AoA, lexical proficiency, etc. are constant across groups), or to the extent that they are not, suggesting avenues for future research. Finally, the present study has implications for bilinguals with native or native-like proficiency in more than one language. Heritage speakers of Spanish make up a significant proportion of the US population; 13.4% of the population aged 5 years or older is currently estimated to speak Spanish at home (U.S. Census Bureau, 2019). Understanding the extent to which language processing behavior in this population is comparable to that of monolingual native English speakers is not only essential to ensuring best practices in clinical and policy decisions, it also promises to enrich our understanding of the basic mechanisms governing language acquisition, speech perception, and cognitive adaptability.

## Materials and Methods

### Participants

Participant recruitment took place *via* Prolific.^1^ The study advertisement was shown only to Prolific users who met the following screening criteria: age 18–35, born in the United States, currently living in the United States, and reporting English as (one of) their native language(s). To identify potential monolingual participants, the following additional criteria were also applied: self-reported fluency in English only and raised in a monolingual environment (“I was raised with my native language only.”). To identify potential L2 Spanish speakers, we screened for participants who reported being fluent in one or more languages in addition to English, were raised in a monolingual environment, and also reported being fluent in Spanish. Finally, to identify potential heritage speakers of Spanish, the following additional screening criteria were applied: US citizen, fluent speaker of one language in addition to English, raised with two or more languages spoken in the home, and fluent in Spanish.

Potential participants identified by these Prolific-internal screening procedures first participated in a study-specific screening session in which they completed the English LexTALE (Lemhöfer and Broersma, 2012), Spanish LexTALE (Izura et al., 2014), and the LEAP-Q (Marian et al., 2007). The LexTALE is a brief (3–4 min) lexical decision task, independently created and normed for each language, providing an objective measure of vocabulary knowledge. The measure used in all LexTALE analyses was the “Average Percent Correct” (Lemhöfer and Broersma, 2012), the grand mean of the average percent correct on word trials and nonword trials. Monolinguals were invited to participate in the main study if they reported Spanish comprehension ability of 3 or less on a scale of 0 (no knowledge) to 10 (like a native speaker), while L2 and heritage participants were initially invited to participate if they reported a 7 or higher. Following an initial period of recruitment (20–25 participants per group), only bilingual participants who achieved at least 60% on the Spanish LexTALE were invited to participate, in order to facilitate regression-based analyses involving this variable, with a final target group size of around 30 per group.

A total of 101 participants completed the main experiment. All reported normal hearing and no history of speech or language disorder. Ten participants were excluded due to low effort responses in the experimental task (defined as either no response or response times faster than 300 ms for more than 25% of trials), one was excluded due to ambiguous responses on the LEAP-Q, and one was excluded for being an early English-Mandarin bilingual, leaving a total of 89 participants’ data for analysis. The LEAP-Q and LexTALE data for the final sample of participants are summarized in Table 1. Several heritage participants had missing data for the question concerning how many years they had spent in an English- (five participants) or Spanish-speaking (three participants) household. The latter three participants were excluded from the individual differences analyses but were included in the group analyses because they listed Spanish as a language spoken in their home growing up.

**Table 1:** Summary of participant characteristics (means and *SD*s).

	Monolingual English		L2 Spanish		Heritage Spanish	
N (N female)	28 (12 F)		30 (18 F)		31 (12 F)	
Age	28.0 (5.1)		**27.9 (5.0)**		**24.6 (4.7)**	
	Eng	Spa	Eng	Spa	Eng	Spa
Self-rated comprehension	10.0 (0.2)	0.5 (0.6)	10.0 (0)	**6.9 (2.0)**	10.0 (0)	**9.0 (1.4)**
Self-rated speaking	10.0 (0)	0.2 (0.4)	10.0 (0.2)	**6.4 (1.8)**	9.9 (0.4)	**7.5 (1.6)**
Self-rated reading	10.0 (0.2)	0.3 (0.6)	10.0 (0)	7.1 (2.1)	10.0 (0)	7.3 (2.2)
Self-ratings composite	10.0 (0.1)	0.4 (0.5)	10.0 (0.1)	**6.8 (1.7)**	10.0 (0.1)	**7.9 (1.4)**
Age of acquisition	0.2 (0.7)	12.6^*^ (3.0)	**0.1 (0.4)**	**13.1 (5.1)**	**1.5 (2.2)**	**0.7 (1.4)**
# yrs. in household where this is spoken^**^	28.0 (5.1)	0 (0)	**27.0 (5.1)**	**1.1 (2.1)**	**19.8 (10.7)**	**21.1 (6.4)**
Percent daily exposure^***^	93.5 (9.6)	3.8 (7.4)	**84.2 (15.9)**	**12.9 (13.6)**	**70.5 (15.6)**	**24.6 (13.6)**
LexTALE	95.0 (4.7)	47.6 (5.9)	94.4 (6.2)	59.1 (6.6)	91.2 (7.6)	65.1 (15.7)

Bolded cells are those for which means for the two bilingual groups differed (adjusted *α* = 0.017). See text for more detailed comparisons.

* Average for participants who entered a response (*n* = 10).

** See note in the text regarding missing data.

*** Some participants did not give percentages that summed to 100; these were rescaled to add up to 100.

The three participant groups were compared using one-way ANOVAs. These returned differences in age [*F*(2,86) = 4.7, *p* = 0.01], English age of acquisition [*F*(2,86) = 10.9, *p* < 0.001], daily exposure to English [*F*(2,86) = 19.9, *p* < 0.001], number of years in an English-speaking household [*F*(2,81) = 10.0, *p* < 0.001], and all of the measures related to Spanish experience (all *F*s > 20.0, all *p*s < 0.001). There was a marginal difference in English LexTALE scores [*F*(2,86) = 3.1, *p* = 0.05]. Two-tailed Welch-corrected *t*-tests with a Bonferroni-adjusted *α* of 0.017 were used to determine which pairwise group comparisons were significant. For age, the Heritage group was slightly younger than both the Monolingual [*t*(55.0) = −2.6, *p* = 0.01] and L2 group [*t*(58.5) = −2.8, *p* = 0.009]. For the English measures, the Monolingual and L2 group differed only in their daily exposure to English [*t*(48.1) = −2.7, *p* = 0.009]. The Heritage group reported a later English AoA than both the Monolingual [*t*(36.2) = 3.5, *p* = 0.002] and the L2 [*t*(32.1) = 3.6, *p* < 0.001] groups, as well as fewer years spent in an English-speaking household and less daily English exposure than both the Monolingual [*t*(35.3) = −3.5, *p* = 0.001; *t*(50.5) = −6.9, *p* < 0.001] and L2 groups [*t*(34.8) = −3.1, *p* = 0.004; *t*(58.8) = −3.3, *p* = 0.001]. The Heritage group’s English LexTALE scores were marginally lower than that of the Monolinguals [*t*(50.9) = −2.3, *p* = 0.02] and the L2 group [*t*(57.4) = −1.8, *p* = 0.08]. For all of the Spanish measures, both bilingual groups differed significantly from the Monolinguals (all |*t*|*s* > 2.7, all *p*s < 0.01), with the exception of Spanish AoA, where the L2 group did not differ from the subset of 10 Monolinguals who had studied Spanish [*t*(27.6) = −0.38, *p* = 0.71], but the Heritage group did [*t*(10.3) = 12.3, *p* < 0.001]. The two bilingual groups differed from one another for all Spanish measures (all |*t*|*s* > 2.5, all *p*s < 0.016) with the exception of self-rated reading [*t*(59.0) = 0.4, *p* = 0.69] and Spanish LexTALE scores; the latter comparison was marginal [*t*(40.6) = 2.0, *p* = 0.06]. No other differences approached significance.

The monolingual group was on the whole quite monolingual. Just 10 of 28 reported having studied Spanish in school, with an average AoA of 12.6 (*SD* = 3.0) and an average composite Spanish self-rating of 0.4 (*SD* = 0.5), while 10 of 28 reported experience with a language other than Spanish, with an average AoA of 15.6 (*SD* = 4.9) and an average composite self-rating of 1.9 (*SD* = 1.7).

### Procedure

#### Experimental Session

All experimental procedures were conducted using Gorilla Experiment Builder^2^ (Anwyl-Irvine et al., 2020), and participants completed them *via* the Internet in a location of their choosing. Participation was restricted to users of desktop computers rather than mobile devices to maximize the probability that participants would be seated, in a location with minimal distractions.

The experiment reported here was the second experimental task of the session. After giving informed consent, participants completed a six-trial headphone check (Woods et al., 2017). The first experimental task in the session was a word transcription task, followed by the lexical decision task reported here, followed by a phonetic perception task, and followed finally by the AX variant of the Continuous Performance Task (Braver et al., 2001; Morales et al., 2013). The full session took approximately 90 min, and participants were compensated $15 for their time with a $1 bonus for successfully completing all tasks in the session.

#### Design

The lexical decision task comprised a total of 240 trials, divided into four blocks of 60 trials each, with six practice trials at the beginning of each block and an opportunity to take a short break in between blocks. Each block consisted of half real English words and half nonwords derived from English words, and the 30 word trials in each block consisted of half English-Spanish cognates and half non-cognates (see “Materials”). Each of the four blocks constituted a different noise condition: the first block was completed in the clear (i.e., no background noise), followed by a block with speech-shaped noise (SSN), a block with English two-talker babble (E2TB), and finally a block with Spanish two-talker babble (S2TB). Block order was fixed to keep any ordering effects constant across participants rather than further complicate the design. The E2TB block was ordered before the S2TB block to prevent any carryover effects of cross-language activation from one block to the next (Misra et al., 2012). The full set of stimuli was divided into four sets, with the cognates and non-cognates within each set matched as closely as possible (see “Materials”). Four different versions of the experiment were created, rotating the item sets through the different noise conditions, to ensure that any cognate effects were not dependent on the specific items in a given condition. Trial order was fully randomized within each block.

#### Trial Procedure

On each trial, participants heard a single word or nonword embedded in the carrier phrase, “Now I’ll say…” and were asked to “determine whether the last item in the sentence is a real English word or a made-up word.” Participants were asked to use their left hand to press “1” on their computer keyboard to respond “real word” or their right hand to press “0” to respond “not a word.” The response options were displayed on the screen throughout the task. For trials in the noise blocks, the noise started 500 ms before the onset of the carrier phrase and continued until 500 ms after the offset of the target word. Participants had up to 3,000 ms following the onset of the target word to make a response, at which point the words “Time’s up! Try to respond faster!” were displayed on the screen.

### Materials

The full list of experimental stimuli is available at https://osf.io/t9prb. The stimuli were compiled by two research assistants with language backgrounds equivalent to the bilingual participants in the experiment (both native English, proficient in Spanish, one a late L2 learner, and one a heritage speaker of Spanish), under the supervision of the author. All stimuli were two syllables long, with stress overwhelmingly on the first syllable. The nonwords were loosely based on the real words and were created by altering two or more phonemes of each word stimulus so as to obscure the relationship between the word and its derived nonword. For example, the nonword *reckle* was derived from the target word *metal*. None of the target words appeared within the same block as their derived nonword.

Cognate and noncognate stimuli were matched on the following attributes (see Table 2; all statistics obtained from the CLEARPOND lexical database; Marian et al., 2012): log-transformed word frequency (from the SUBTLEX-US corpus; Brysbaert and New, 2009), length in phonemes, number of English and Spanish phonological neighbors (most words had no Spanish phonological neighbors), and the mean positional frequency and biphone frequency of all phonemes/biphones in the word. Two-tailed Welch-corrected *t*-tests comparing cognates to noncognates for each of the four stimulus subsets separately, and the full stimulus set combined, confirmed that stimuli did not differ along any of these dimensions (all |*t*|*s* < 1.7, all *p*s > 0.10). However, two-tailed continuity-corrected Wilcoxon tests examining word durations indicated that cognate stimuli were overall longer than noncognates (*W* = 2,354, *p* = 0.004); this difference was also significant for Set 1 (*W* = 164, *p* = 0.03) and marginal for Set 2 (*W* = 160, *p* = 0.05).

**Table 2:** Summary of stimulus characteristics (means and *SD*s, real words only).

	Set 1		Set 2		Set 3		Set 4		All	
	Cog (***n*** = 15)	Non (***n*** = 15)	Cog (***n*** = 15)	Non (***n*** = 15)	Cog (***n*** = 15)	Non (***n*** = 15)	Cog (***n*** = 15)	Non (***n*** = 15)	Cog (***n*** = 60)	Non (***n*** = 60)
log(Freq)	1.7 (0.4)	1.7 (0.2)	1.7 (0.3)	1.7 (0.2)	1.7 (0.4)	1.7 (0.2)	1.8 (0.4)	1.8 (0.3)	1.7 (0.4)	1.7 (0.2)
									*t*(103.4) = −0.18, *p* = 0.86	
Length in phonemes	5.0 (1.2)	5.1 (0.8)	5.1 (0.9)	4.8 (1.3)	4.6 (0.8)	5.0 (1.3)	5.3 (1.1)	5.2 (1.1)	5.0 (1.0)	5.0 (1.1)
									*t*(117.1) = −0.17, *p* = 0.87	
Eng phon nbors	4.3 (4.5)	4.5 (4.7)	5.1 (5.6)	5.1 (4.7)	4.5 (3.9)	4.5 (5.0)	4.9 (5.1)	3.9 (4.7)	4.7 (4.7)	4.5 (4.7)
									*t*(118.0) = 0.19, *p* = 0.85	
Spa phon nbors	0.00 (0.0)	0.00 (0.00)	0.20 (0.77)	0.00 (0.00)	0.00 (0.00)	0.07 (0.26)	0.13 (0.52)	0.00 (0.00)	0.08 (0.46)	0.02 (0.13)
									*t*(68.2) = 1.1, *p* = 0.29	
Mean phon freq	0.04 (0.02)	0.05 (0.01)	0.05 (0.02)	0.05 (0.01)	0.05 (0.02)	0.05 (0.01)	0.05 (0.02)	0.05 (0.02)	0.05 (0.02)	0.05 (0.01)
									*t*(113.4) = −0.23, *p* = 0.82	
Mean biphon freq	0.006 (0.004)	0.006 (0.003)	0.005 (0.004)	0.004 (0.004)	0.005 (0.004)	0.004 (0.002)	0.006 (0.003)	0.006 (0.005)	0.005 (0.004)	0.005 (0.004)
									*t*(118.0) = 0.51, *p* = 0.61	
Duration in ms	712 (143)	615 (94)	738 (91)	663 (174)	670 (119)	655 (159)	706 (131)	668 (147)	707 (122)	651 (144)
	*W* = 164, *p* = 0.03		*W* = 160, *p* = 0.05		*W* = 126, *p* = 0.59		*W* = 132, *p* = 0.44		*W* = 2,354, *p* = 0.004	

Cognate vs. noncognate means did not differ for word frequency, length in phonemes, number of English or Spanish neighbors, or mean positional or biphone frequency (all |*t*|*s* < 1.7, all *ps* > 0.10), but Wilcoxon tests indicated stimulus durations were longer for cognates overall and in Set 1, and marginally so in Set 2.

The stimuli were recorded by a native speaker of Central American Spanish whose English proficiency and accent were subjectively native-like. This speaker was chosen for her ability to record native-sounding stimuli for both the English and Spanish versions of the experiments. The recordings were made in a sound-attenuated booth at a 44.1 kHz sampling rate and 16-bit depth. The stimuli were later downsampled to 22,050 Hz and converted to .mp3 format to minimize loading delays over the internet.

Speech-shaped noise was created by taking the long-term average spectrum of the files used to create the babble noise and then using the spectral shape as a filter for white noise; this was done with a Praat script derived from code used by Quené and Van Delft (2010). English and Spanish two-talker babble were created using freely available news podcasts. This allowed the choice of voices and accents while controlling for register and subject material across languages. Four podcasts were chosen, all with female presenters speaking a standard, not obviously regionally specific variety of the language. All non-speech noise, speech produced by a talker other than the main newscaster and pauses longer than 500 ms were manually edited out. A random selection from each of the two English podcasts was then combined with each clip of target speech to make the English two-talker babble, and similarly for Spanish, using a Praat script (Boersma and Weenink, 2020).

The root mean square (RMS) amplitude for each clip of target speech was scaled to 70 dB SPL, as was the noise, for a signal-to-noise ratio of 0 dB. This ratio was chosen based on informal pilot testing and on findings in the literature suggesting that this level would be challenging but feasible for a range of participant profiles (Garcia Lecumberri et al., 2010).

## Analysis and Results

### Overview of Analysis Procedures

All statistical analyses were carried out in R (R Core Team, 2020; version 4.0.3) using the following packages: lme4 (Bates et al., 2015; version 1.1.26), lmerTest (Kuznetsova et al., 2017; version 3.1.3), ggplot2 (Wickham, 2016; version 3.3.3), emmeans (Lenth, 2021; version 1.6.3), sjPlot (Lüdecke, 2021; version 2.8.9), ggbiplot (Vu, 2011; version 0.55), and ggeffects (Lüdecke, 2018). Numerical predictors were centered and scaled, and transformed when appropriate, as determined by visual inspection of q-q plots. In general, the maximal random effects structure that would converge was used (Barr et al., 2013); more details regarding model selection are given below.

The first analysis asked whether the three participant groups showed differential effects of Cognate Status, Noise Condition, or their interaction. Unfortunately, due to model convergence issues (perhaps the result of quasi-separation; Kimball et al., 2019), it was not possible to fit a sufficiently complex mixed effects logistic regression to the accuracy data. We therefore present descriptive statistics for both real words and nonwords in order to qualitatively evaluate response strategies across groups and conditions. For example, particularly low nonword accuracy could indicate that some listeners were more likely to default to a “real word” response, potentially making it inappropriate to compare RTs across groups.

For the RT analysis, Participant Group was Helmert coded such that the first contrast compares the two bilingual groups to one another, and the second contrast compares the average of the bilingual groups to the monolinguals; any apparent cognate effects for the monolinguals are likely due to durational differences in the stimuli, so it is important to demonstrate that the bilinguals differ from the monolinguals. Stimulus Duration and its interactions with Noise Condition and Participant Group were also included as covariates. Both Cognate Status and Noise Condition were contrast coded; the coefficient for Cognate Status corresponds to the overall difference between cognates and noncognates, and the coefficients for Noise Condition provide comparisons of the following conditions: (1) Clear vs. all three noise conditions, indexing the overall recognition deficit in noise, (2) Speech-Shaped Noise vs. the average of English and Spanish Two-Talker Babble, indexing whether the groups responded differently to energetic vs. informational masking, and (3) English Two-Talker Babble vs. Spanish Two-Talker Babble, indexing whether the effect of informational masking differed by masking language. The interaction terms involving Cognate Status, Participant Group, and Noise Condition thus ask whether the magnitude of any cognate effects was modulated by language background or masking noise.

The second set of analyses examined individual differences in RTs among the bilingual participants, treating language experience-related predictors as continuous rather than categorical, as has recently been advocated in the literature (Luk and Bialystok, 2013; Fricke et al., 2019). Principal component analysis was used to derive orthogonal measures of language experience, and model comparisons were used to determine the best-fitting model incorporating these measures.

### Group Analyses

#### Descriptive Statistics for Word Recognition Accuracy

Trials with RTs faster than 300 ms were removed from the dataset (0.7% of the data). The cut-off point for long RTs was the one imposed by the experimental procedure (3,000 ms).

Table 3 gives descriptive statistics for the accuracy data, first broken down more globally in terms of lexical status and the overall recognition deficit in noise (top portion), and second more granularly in terms of cognate status and noise condition (bottom portion). Accuracy for nonword stimuli can be taken as an index of word bias, with lower nonword accuracy indicating a greater tendency to default to a “real word” response.

**Table 3:** Descriptive statistics for accuracy (proportion correct trials and *SD*s) by condition and participant group.

	Monolingual English			L2 Spanish			Heritage Spanish		
	Nonwords	Words		Nonwords	Words		Nonwords	Words	
Overall accuracy (Clear)	0.94 (0.06)	0.97 (0.03)		0.92 (0.10)	0.96 (0.03)		0.89 (0.12)	0.98 (0.03)	
Overall accuracy (all noise)	0.77 (0.09)	0.79 (0.09)		0.73 (0.13)	0.82 (0.08)		0.75 (0.13)	0.75 (0.11)	
	**Nonwords**	**Cog**	**Noncog**	**Nonwords**	**Cog**	**Noncog**	**Nonwords**	**Cog**	**Noncog**
Clear	0.94 (0.06)	0.97 (0.05)	0.98 (0.03)	0.92 (0.10)	0.96 (0.05)	0.97 (0.04)	0.89 (0.12)	0.97 (0.04)	0.98 (0.04)
SSN	0.78 (0.12)	0.82 (0.11)	0.73 (0.12)	0.70 (0.15)	0.84 (0.11)	0.78 (0.14)	0.73 (0.14)	0.79 (0.13)	0.67 (0.14)
E2TB	0.77 (0.16)	0.80 (0.14)	0.79 (0.13)	0.76 (0.12)	0.83 (0.12)	0.77 (0.12)	0.75 (0.20)	0.79 (0.16)	0.77 (0.17)
S2TB	0.76 (0.12)	0.82 (0.19)	0.77 (0.18)	0.75 (0.15)	0.87 (0.10)	0.82 (0.13)	0.76 (0.16)	0.77 (0.18)	0.74 (0.21)

There are several points to note. First, in the clear, word accuracy was at ceiling for all three participant groups at around 97%. Nonword accuracy was generally high but varied more than word accuracy: nonword accuracy for heritage bilinguals was 89%, versus 92% for L2 bilinguals and 94% for monolinguals. However, differences in both word and nonword accuracy were within 1 *SD* across groups.

With respect to masking effects, the three groups showed a comparable drop-off in word recognition accuracy of around 15–20%, with all groups again within a single *SD*. The noise deficit was numerically greatest for the heritage bilinguals, at 23% averaged across noise and cognate conditions. For all three groups, word biases increased considerably in noise, with nonword accuracy decreasing around 15–20% when averaged across all noise conditions. The numerically lowest nonword accuracy was found for the L2 group in speech-shaped noise, at just 70%, though again this was within 1 *SD* of the other groups.

On average, cognates were recognized more accurately than noncognates in noise, by monolinguals as well as bilinguals, consistent with a slight potential benefit for longer stimuli in noise for all participant groups.

##### Discussion of Accuracy Data

Word recognition accuracy suffered considerably in noise, and word biases increased substantially, with comparable effects across noise types and participant groups. In general, then, the three participant groups employed qualitatively similar response criteria and adjusted their response criteria in similar ways. Importantly, cognates tended to be recognized more accurately than noncognates, irrespective of participant language background. This suggests that the partial confound of stimulus duration and cognate status may be of concern and that the statistical model examining RTs should take this into account.

#### Response Time Analysis

##### Data Preparation and Model Fitting Procedure

Response times (RTs) were measured from the onset of the target word and were log-transformed prior to analysis. All nonword trials, RTs faster than 300 ms, and incorrect responses (17% of the remaining data) were removed from the dataset. We then removed any responses that were more than two standard deviations from each participant’s mean RT (4% of the remaining data). This data cleaning procedure left a total of 8,200 data points for analysis.

A model was fit that included all of the experimentally manipulated variables (Participant Group, Noise Condition, and Cognate Status) and their two- and three-way interactions as fixed effects, plus fixed effects of Stimulus Duration, and its two-way interactions with Participant Group and Noise Condition, plus the maximal random effects structure that would converge; this included by-participant and by-item random intercepts and slopes for the effect of Noise Condition.

##### RT Results

**Figure 1:**
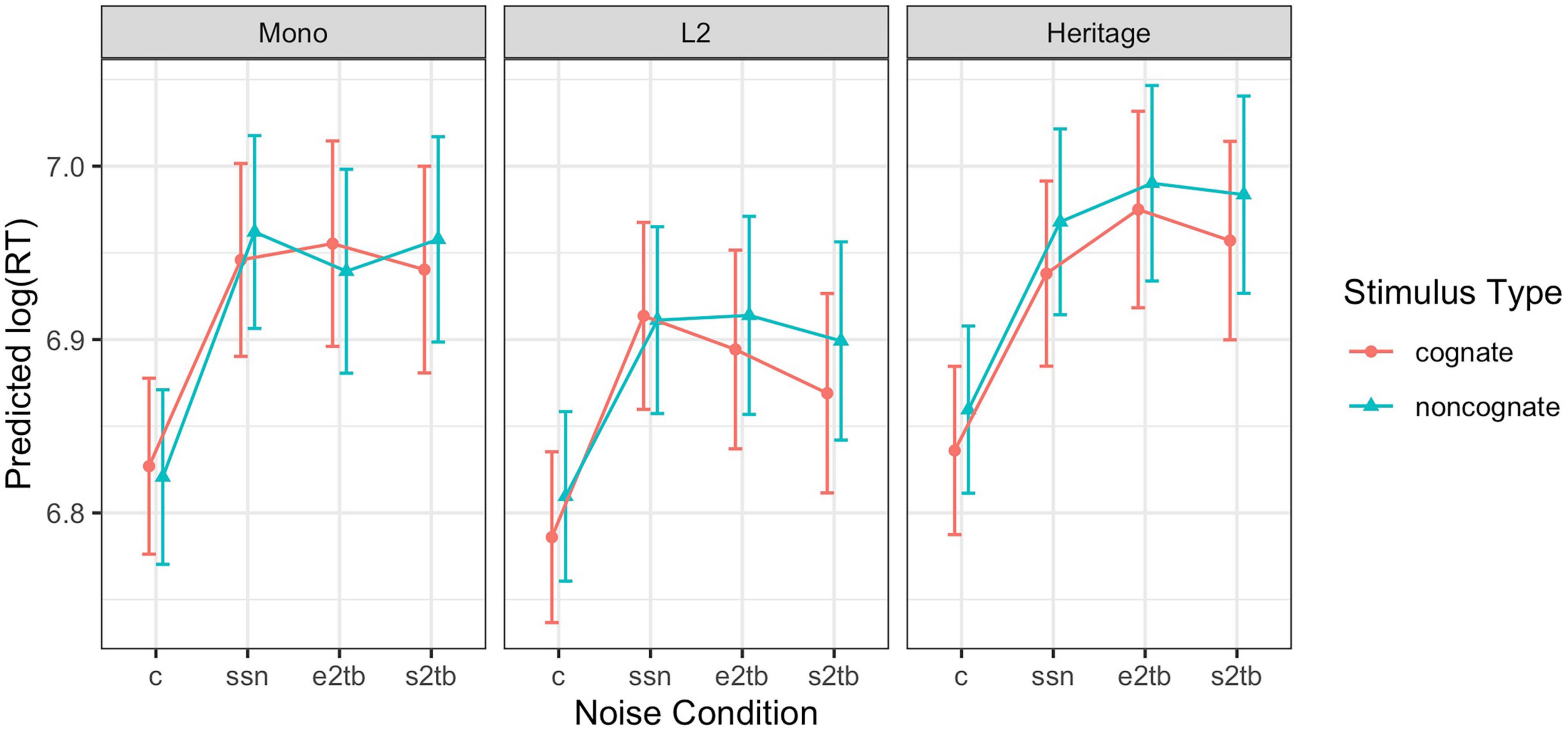
Predicted response times (estimated marginal means with prediction intervals) for correct word trials in the lexical decision task across stimulus types, noise conditions, and participant groups, using the fitted model from Table 4. (c = clear, ssn = speech-shaped noise, e2tb = English two-talker babble, and s2tb = Spanish two-talker babble).

Figure 1 shows the predicted RT values for the fitted model, i.e., with effects of stimulus duration partialled out, and includes prediction intervals as implemented in the ggeffects R package. The model is given in Table 4, with coefficients numerically labeled for ease of reference.

**Table 4:** Fixed and random effects for the model comparing RTs across participant groups.

Label	Predictor	Fixed effect estimates			
		Estimate	*SE*	*CI* (95%)	*p*
	(Intercept)	6.91	0.01	6.88–6.94	**<0.001**
(1)	Stimulus duration	0.07	0.01	0.06–0.09	**<0.001**
(2)	Noise Contrast 1 (Clear vs. All Noise)	−0.12	0.01	−0.14–−0.10	**<0.001**
	Noise Contrast 2 (SSN vs. 2 TB average)	0.00	0.01	−0.03–0.03	0.989
	Noise Contrast 3 (E2TB vs. S2TB)	0.01	0.01	−0.01–0.03	0.287
(3)	Group Contrast 1 (L2 vs. Heritage)	0.03	0.02	0.00–0.06	**0.034**
	Group Contrast 2 (Monoling vs. Biling)	0.00	0.01	−0.01–0.02	0.654
	Cognate status	0.01	0.01	−0.01–0.04	0.302
(4)	StimDur * NoiseC1	0.02	0.01	0.01–0.03	**0.003**
	StimDur * NoiseC2	−0.00	0.01	−0.02–0.01	0.770
	StimDur * NoiseC3	−0.01	0.01	−0.02–0.01	0.320
(5)	StimDur * GroupC1	−0.00	0.00	−0.01–−0.00	**0.021**
	StimDur * GroupC2	0.00	0.00	−0.00–0.00	0.160
	NoiseC1 * CogStatus	-0.00	0.01	−0.03–0.02	0.904
	NoiseC2 * CogStatus	−0.00	0.01	−0.03–0.03	0.946
	NoiseC3 * CogStatus	−0.02	0.01	−0.05–0.01	0.208
	GroupC1 * CogStatus	0.00	0.00	−0.01–0.01	0.481
(6)	GroupC2 * CogStatus	−0.01	0.00	−0.01–−0.00	**0.019**
	NoiseC1 * GroupC1	−0.01	0.01	−0.03–0.01	0.429
	NoiseC2 * GroupC1	−0.02	0.02	−0.05–0.01	0.180
	NoiseC3 * GroupC1	−0.00	0.01	−0.02–0.02	0.694
	NoiseC1 * GroupC2	−0.00	0.01	−0.02–0.01	0.479
	NoiseC2 * GroupC2	0.00	0.01	−0.02–0.02	0.763
	NoiseC3 * GroupC2	−0.01	0.01	−0.02–0.01	0.311
	NoiseC1 * GroupC1 * CogStatus	−0.00	0.01	−0.02–0.01	0.655
	NoiseC2 * GroupC1 * CogStatus	0.02	0.01	−0.00–0.04	0.086
	NoiseC3 * GroupC1 * CogStatus	−0.00	0.01	−0.02–0.02	0.972
	NoiseC1 * GroupC2 * CogStatus	−0.01	0.01	−0.02–0.01	0.321
	NoiseC2 * GroupC2 * CogStatus	0.01	0.01	−0.00–0.02	0.193
	NoiseC3 * GroupC2 * CogStatus	−0.01	0.01	−0.02–0.01	0.300
**Random effects**					
	*σ* ^2^	0.02			
	*τ*_00 Stimulus_	0.01			
	*τ*_00 Participant_	0.01			
	*τ*_11 Stimulus.NoiseC1_	0.00			
	*τ*_11 Stimulus.NoiseC2_	0.00			
	*τ*_11 Stimulus.NoiseC3_	0.00			
	*τ*_11 Participant.NoiseC1_	0.01			
	*τ*_11 Participant.NoiseC2_	0.01			
	*τ*_11 Participant.NoiseC3_	0.00			
	Observations	8,200			
	Marginal R^2^/Conditional R^2^	0.162/0.584			

Significant fixed effects are labeled for ease of reference within the text.

In terms of main effects, Stimulus Duration was highly predictive of RT (1); longer stimuli elicited longer RTs. The Clear vs. All Noise comparison was significant (2) such that RTs in noise were slower than in the clear, and the L2 vs. Heritage listeners comparison was significant (3) such that RTs were faster for L2 as compared to Heritage listeners.

**Figure 2:**
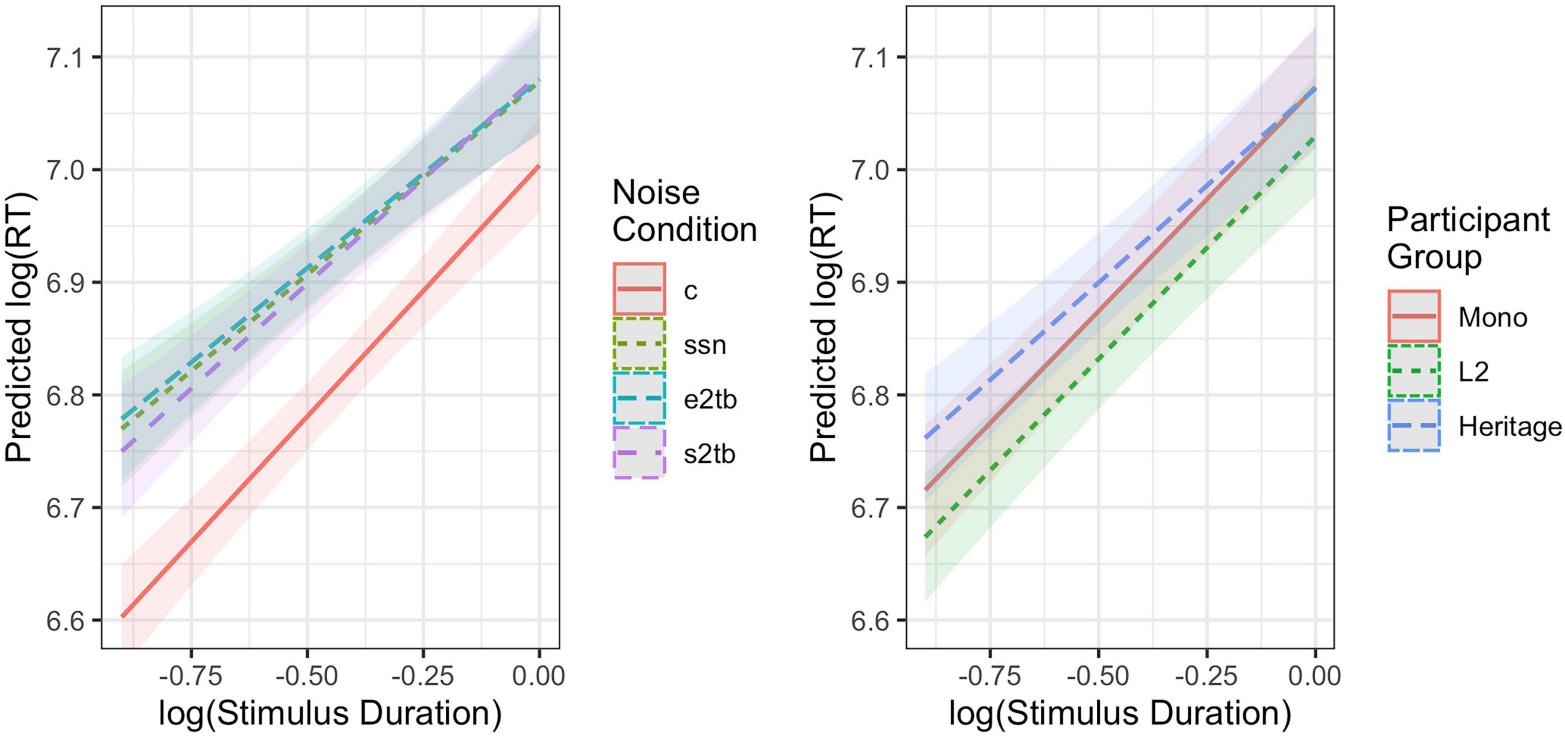
Predicted response times (estimated marginal trends with prediction intervals) showing effects of stimulus duration across noise conditions and participant groups, using the fitted model from Table 4. (c = clear, ssn = speech-shaped noise, e2tb = English two-talker babble, and s2tb = Spanish two-talker babble).

There were two two-way interactions involving Stimulus Duration, which are plotted as marginal effects in Figure 2. Stimulus Duration interacted with Noise Condition (4); the lengthening effect of longer duration on RTs was attenuated in noise as compared to in the clear. This was driven by disproportionately slower RTs to the shortest stimuli in noise (left panel). Stimulus Duration also interacted with Group (5); the lengthening effect of longer duration was attenuated for the Heritage listeners as compared to the L2 group. This was likewise driven by slower RTs to the shortest stimuli (right panel).

There was no main effect of Cognate Status, but the coefficient for Cognate Status differed for Bilinguals as compared to Monolinguals (6). Comparison of the estimated marginal means showed that the magnitude of the cognate effect was greater for Bilinguals as compared to Monolinguals (estimate = 0.018, SE = 0.008, *p* = 0.02). For Monolinguals for both cognates and noncognates, and for Bilinguals for noncognates only, the estimated mean log RT was 6.92; for Bilinguals for cognates, the mean was 6.90, corresponding to a cognate facilitation effect of about 20 ms, all else being equal. Cognate Status did not enter into any additional interactions, indicating that the magnitude of the cognate effect was not modulated by the type of noise.

#### Interim Discussion for RT Analyses

All listener groups responded more slowly in noise as compared to in the clear, and the overall degree of slowing was consistent across groups. While the former is expected, the latter is somewhat surprising in light of previous findings concerning heritage listeners’ word recognition in noise (Mayo et al., 1997; Rogers et al., 2006; Morini and Newman, 2020). We consider this finding in more detail in the General Discussion.

The effects of cognate status on RT were surprisingly straightforward, though not as predicted. After statistically controlling for differences in stimulus duration, cognate facilitation was small in magnitude but significant for bilinguals as compared to monolinguals. Crucially, there was no evidence that the magnitude of facilitation was affected by the presence or type of competing noise. Contrary to what has been assumed in the literature, then, the results do not support the idea that cross-language activation is greater in noise relative to in the clear, at least during word recognition in the dominant language, a point we return to in the General Discussion. We next turn to the question of whether language experience modulated either the cognate effect or the noise masking effects.

### Individual Differences Analysis

#### Overview of Individual Differences Analysis Procedure

The individual differences analysis asks whether differential experience in English vs. Spanish affects English word recognition, so we restrict our attention to the two bilingual groups. We focus on response times; the accuracy data suggested that the three groups employed similar response strategies, and the group analysis provided evidence that the RT model was able to adequately statistically control for the durational differences between stimulus types.

Seven language experience-related predictors were considered: composite self-ratings for Spanish proficiency (i.e., the averaged self-ratings for Spanish comprehension, speaking, and reading ability), Spanish age of acquisition, number of years in a Spanish-speaking household, self-reported current daily exposure to English and Spanish, and LexTALE scores in English and Spanish. English self-ratings, AoA, and years of household exposure were at or near ceiling, so they were not considered. Table 5 gives Kendall’s rank correlation coefficients for these seven variables; q-q plots indicated that all were non-normally distributed to some extent. In general, the questionnaire responses were moderately intercorrelated, while the LexTALE scores were less correlated both with each other and with the self-report measures. To mitigate multicollinearity, principal component analysis was used to derive orthogonal indices of language experience.

**Table 5:** Correlation matrix (Kendall’s *τ*) for individual difference measures.

	Spa Composite Self-Rating	Spa AoA	# Yrs in Spa Household	% Daily Eng Exposure	% Daily Spa Exposure	Eng LexTALE
Spa AoA	−0.23, *p* = 0.08 *p* adj. *=* 0.81					
# Yrs in Spa Household	0.26, *p* = 0.05 *p* adj. *=* 0.65	−0.55, *p* = 0.00 *p* adj. *=* 0.00				
% Daily Eng	−0.31, *p* = 0.02 *p* adj. *=* 0.33	0.28, *p* = 0.03 *p* adj. *=* 0.53	−0.28, *p* = 0.03 *p* adj. *=* 0.52			
% Daily Spa	0.27, *p* = 0.04 *p* adj. *=* 0.61	−0.28, *p* = 0.03 *p* adj. *=* 0.53	0.36, *p* = 0.01 *p* adj. *=* 0.11	−0.83, *p* = 0.00 *p* adj. *=* 0.00		
Eng LexTALE	−0.01, *p* = 0.95 *p* adj. *=* 1.0	0.15, *p* = 0.26 *p* adj. *=* 1.0	−0.06, *p* = 0.65 *p* adj. *=* 1.0	0.25, *p* = 0.06 *p* adj. *=* 0.74	−0.20, *p* = 0.14 *p* adj. *=* 1.0	
Spa LexTALE	0.24, *p* = 0.07 *p* adj. *=* 0.76	−0.01, *p* = 0.96 *p* adj. *=* 1.0	0.13, *p* = 0.33 *p* adj. *=* 1.0	−0.17, *p* = 0.20 *p* adj. *=* 1.0	0.14, *p* = 0.31 *p* adj. *=* 1.0	0.18, *p* = 0.18 *p* adj. *=* 1.0

#### Principal Component Analysis

**Figure 3:**
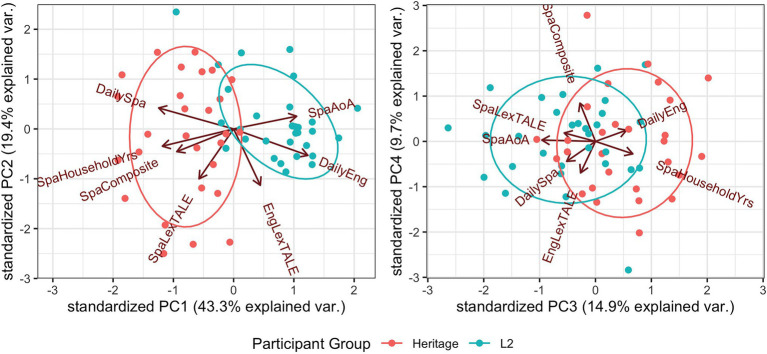
Bilingual participants visualized with respect to language experience principal components 1–4 (*cf.* Table 6).

Principal Component Analysis was applied to the language experience predictors listed above. A Scree plot showed a drop-off in explained variance between the fifth and sixth principal components (PCs), so analysis was restricted to PCs one through five; each of these also accounted for at least 5% of variance, a value sometimes cited as a cut-off (Baayen, 2008). Subsequent analyses showed that PC5 did not predict RTs, so we do not consider it further. Figure 3 plots each bilingual listener as a function of the first four PCs, which account for a total of 87% of the variance in language experience measures. Table 6 gives the variable loadings for PCs 1–4; these correspond to the covariances between the seven original variables and the PCs.

**Table 6:** Variable loadings for PCs 1–4.

	PC1 (“DailySpaExposure”)	PC2 (“VerbalAbilityEng”)	PC3 (“SpaAoA”)	PC4 (“SpaSelfRating”)
SpaComposite	−0.62	−0.30	−0.18	**0.56**
SpaAoA	0.69	0.17	**−0.63**	0.02
SpaHouseholdYrs	**−0.78**	−0.23	**0.44**	−0.18
DailyEng	**0.81**	−0.35	0.37	0.16
DailySpa	**−0.82**	0.28	−0.34	−0.29
EngLexTALE	0.29	**−0.75**	−0.18	**−0.46**
SpaLexTALE	−0.38	**−0.66**	−0.37	0.12

Variables mentioned in the text (i.e., those with highest covariances between variables and components) have been bolded.

PC1 (Figure 3, left panel) largely discriminates between the two bilingual groups. The heritage group is characterized by lower values of PC1, while the L2 group tends to have higher values; note that because the rotation of the PC axes is arbitrary, low values of PC1 correspond primarily to *greater* daily Spanish exposure and *more* years in a Spanish-speaking household. We refer to this dimension as “PC1-DailySpaExposure.”

PC2 (left panel, y-axis) does not separate the participant groups and corresponds most closely to English LexTALE performance, followed by Spanish LexTALE performance. The heritage group shows greater variation along this axis than the L2 group. We refer to this dimension as “PC2-VerbalAbilityEng.”

PC3 (Figure 3, right panel) discriminates somewhat between participant groups and is most strongly associated with Spanish AoA, followed by number of years in a Spanish-speaking household. We refer to this dimension as “PC3-SpaAoA.”

Finally, PC4 (right panel, y-axis) corresponds primarily to Spanish self-ratings, with a smaller contribution from English LexTALE. Like PC2, PC4 does not separate the two bilingual groups, and the heritage group shows greater variation along this axis. We refer to this dimension as “PC4-SpaSelfRatings.”

We note several additional considerations. First, we underscore that participants were overwhelmingly English dominant; all but four had higher self-ratings for English than Spanish, and all but two (different participants) had higher LexTALE scores in English than Spanish. The PCs derived from this analysis therefore index the strength of Spanish experience *relative to other English-dominant bilinguals who participated in the experiment*.

Second, Spanish LexTALE loaded weakly-to-moderately onto PCs 1–4, indicating that Spanish vocabulary scores did not represent a unique axis of variation in language experience for these bilinguals. However, based on previous work (e.g., Kilman et al., 2014; Scharenborg et al., 2018), we were nonetheless interested in the predictive power of objectively measured Spanish knowledge. We therefore included it in the individual differences analyses alongside the PCs. Kendall’s rank correlation tests with Holm-adjusted *p* values indicated that Spanish LexTALE scores were not significantly correlated with any of the PCs.

#### Model Selection Procedure

A two-step model selection procedure was used. First, to determine whether any of the language experience measures predicted word recognition performance, we refit the full model from Table 4 to the bilingual RT data only, with Participant Group recoded as a two-way contrast between L2 and Heritage participants. In Table 7, this is referred to as the “base” model but note that model log likelihood varied depending on the maximal random effects structure that converged for each predictor. We fit four additional, nested models for each predictor: (1) the base model plus a main effect of the predictor, (2) model (1) plus the interaction of the predictor with Participant Group, (3) model (2) plus the interaction of the predictor with either Cognate Status or Noise Condition, and (4) model (3) plus the three-way interaction of the predictor with both Participant Group and Cognate Status/Noise Condition. This nested model evaluation procedure explicitly tests whether either the continuous language experience measures or the binary Group characterization add any predictive power over and above the other.

**Table 7:** Nested comparisons of model log likelihood showing the predictive power of language experience predictors in the first step of the individual differences analysis.

Significant cognate effect model comparisons	logLik	*χ* ^2^	*df*	*p*
Base	665.1			
Base + PC4	665.1	0.0	1	0.993
Base + PC4 + PC4:Group	667.8	5.4	1	0.020
Base + PC4 + PC4:Group + PC4:CogStatus	669.3	2.9	1	0.087
Base + PC4 + PC4:Group + PC4:CogStatus + PC4:CogStatus:Group	671.2	3.9	1	0.048
**Significant noise effect model comparisons**	logLik	***χ*** ^2^	*df*	*p*
Base	1595.0			
Base + PC1	1595.6	1.2	1	0.282
Base + PC1 + PC1:Group	1596.8	2.3	1	0.125
Base + PC1 + PC1:Group + PC1:NoiseCond	1605.2	16.8	3	< 0.001
Base + PC1 + PC1:Group + PC1:NoiseCond + PC1:NoiseCond:Group	1609.9	9.5	3	0.024
Base	1595.0			
Base + PC2	1595.1	0.1	1	0.790
Base + PC2 + PC2:Group	1595.3	0.5	1	0.501
Base + PC2 + PC2:Group + PC2:NoiseCond	1598.5	6.4	3	0.093
Base + PC2 + PC2:Group + PC2:NoiseCond + PC2:NoiseCond:Group	1609.5	22.1	3	< 0.001
Base	1885.0			
Base + PC4	1885.1	0.0	1	0.850
Base + PC4 + PC4:Group	1885.2	0.2	1	0.668
Base + PC4 + PC4:Group + PC4:NoiseCond	1888.7	7.1	3	0.069
Base + PC4 + PC4:Group + PC4:NoiseCond + PC4:NoiseCond:Group	1891.6	5.8	3	0.124
Base	1595.0			
Base + SpaLexTALE	1595.1	0.1	1	0.782
Base + SpaLexTALE + SpaLexTALE:Group	1595.2	0.3	1	0.585
Base + SpaLexTALE + SpaLexTALE:Group + SpaLexTALE:NoiseCond	1603.2	15.9	3	0.001
Base + SpaLexTALE + SpaLexTALE:Group + SpaLexTALE:NoiseCond + SpaLexTALE:NoiseCond:Group	1604.9	3.5	3	0.327

Random effects structures were adjusted following Barr et al. (2013) so that all nested models for a given predictor contained the same random effects. An *α* level of 0.10 was used for model log likelihood comparisons; Matuschek et al. (2017) indicate that *α*_LRT_ = 0.10 is somewhat more conservative than the criterion most commonly used for comparing model AICs, but not so conservative as to overly penalize model complexity. All significant model comparisons from this first step of the analysis procedure are given in Table 7; PCs 1, 2, and 4, and Spanish LexTALE improved model fit.

In the second step, we fit a saturated model consisting of the base model plus the significant predictors from the first step (i.e., PC1-DailySpaExposure, PC2-VerbalAbilityEng, PC4-SpaSelfRating, and Spanish LexTALE) and their two- and three-way interactions with Group and Noise Condition/Cognate Status. Backwards selection using nested model comparisons was then used to optimize model fit and statistical power (Matuschek et al., 2017). Leave-one-out comparisons indicated that all remaining interaction terms were significant at the *α*_LRT_ = 0.10 level (all *p*s < 0.07).

The final, fitted model is given in Table 8, with coefficients numerically labeled for ease of reference. The maximally converging random effects structure contained random by-participant and by-stimulus intercepts. In the text, we report pairwise comparisons for the estimated marginal means at the average ± 1 *SD* of the values of each predictor, with familywise adjustments for *p* values as implemented in the emmeans package.

**Table 8:** Fixed and random effects for the model comparing RTs for bilingual participants only in the individual differences analysis.

Label	Coefficient	Fixed effect estimates			
		Estimate	*SE*	*CI* (95%)	*p*
	(Intercept)	6.87	0.03	6.82–6.93	**<0.001**
(1)	Stimulus duration	0.07	0.01	0.06–0.09	**<0.001**
(2)	Noise Contrast 1 (Clear vs. All Noise)	−0.12	0.01	−0.13–−0.11	**<0.001**
	Noise Contrast 2 (SSN vs. 2 TB average)	0.00	0.01	−0.01–0.02	0.479
	Noise Contrast 3 (E2TB vs. S2TB)	0.01	0.01	−0.00–0.02	0.195
(3)	BilingGroup	0.12	0.06	0.01–0.24	**0.033**
	CogStatus	0.02	0.01	−0.01–0.05	0.180
	PC1-DailySpaExposure	0.05	0.03	−0.02–0.11	0.171
	PC2-VerbalAbilityEng	0.02	0.02	−0.03–0.07	0.419
	PC4-SpaSelfRating	0.01	0.02	−0.03–0.04	0.735
	SpaLexTALE	0.03	0.03	−0.03–0.08	0.335
(4)	StimDur * NoiseC1	0.02	0.01	0.01–0.03	**<0.001**
	StimDur * NoiseC2	−0.01	0.01	−0.02–0.01	0.311
	StimDur * NoiseC3	−0.01	0.01	−0.02–0.00	0.188
(5)	StimDur * BilingGroup	−0.01	0.00	−0.02–−0.00	**0.008**
	NoiseC1 * CogStatus	0.00	0.01	−0.02–0.02	0.931
	NoiseC2 * CogStatus	−0.01	0.01	−0.03–0.02	0.476
	NoiseC3 * CogStatus	−0.02	0.01	−0.04–0.01	0.225
	BilingGroup * CogStatus	0.01	0.01	−0.01–0.03	0.514
(6)	NoiseC1 * BilingGroup	−0.05	0.02	−0.09–−0.01	**0.007**
(7)	NoiseC2 * BilingGroup	−0.08	0.02	−0.13–−0.04	**<0.001**
	NoiseC3 * BilingGroup	−0.02	0.03	−0.07–0.04	0.553
	BilingGroup * PC1	−0.08	0.05	−0.18–0.03	0.139
	NoiseC1 * PC1	−0.02	0.01	−0.04–0.00	0.101
(8)	NoiseC2 * PC1	−0.03	0.01	−0.06–−0.01	**0.012**
	NoiseC3 * PC1	-0.01	0.01	−0.04–0.02	0.581
	BilingGroup * PC2	0.01	0.04	−0.06–0.09	0.792
	NoiseC1 * PC2	0.00	0.01	−0.01–0.02	0.696
	NoiseC2 * PC2	−0.02	0.01	−0.04–0.00	0.066
	NoiseC3 * PC2	−0.01	0.01	−0.03–0.01	0.373
	BilingGroup * PC4	−0.01	0.03	−0.07–0.06	0.834
(9)	NoiseC1 * PC4	−0.02	0.01	−0.03–−0.01	**<0.001**
(10)	NoiseC2 * PC4	−0.04	0.01	−0.05–−0.03	**<0.001**
	NoiseC3 * PC4	0.01	0.01	−0.00–0.03	0.094
	NoiseC1 * SpaLexTALE	−0.01	0.01	−0.02–0.01	0.545
	NoiseC2 * SpaLexTALE	−0.02	0.01	−0.04–0.01	0.171
(11)	NoiseC3 * SpaLexTALE	−0.04	0.01	−0.06–−0.01	**0.002**
(12)	CogStatus * PC4	0.01	0.00	−0.00–0.02	0.070
	NoiseC1 * BilingGroup * CogStatus	−0.02	0.02	−0.06–0.02	0.382
	NoiseC2 * BilingGroup * CogStatus	0.04	0.02	−0.01–0.08	0.131
	NoiseC3 * BilingGroup * CogStatus	−0.00	0.03	−0.06–0.05	0.861
(13)	(NoiseC1 * BilingGroup) * PC2	−0.05	0.01	−0.07–−0.02	**<0.001**
	(NoiseC2 * BilingGroup) * PC2	0.00	0.01	−0.03–0.03	0.936
	(NoiseC3 * BilingGroup) * PC2	0.01	0.02	−0.03–0.04	0.726
(14)	(NoiseC1 * BilingGroup) * PC4	−0.03	0.01	−0.06–−0.01	**0.003**
(15)	(NoiseC2 * BilingGroup) * PC4	−0.03	0.01	−0.05–−0.00	**0.047**
(16)	(NoiseC3 * BilingGroup) * PC4	0.04	0.02	0.01–0.07	**0.004**
(17)	(BilingGroup * CogStatus) * PC4	0.02	0.01	-0.00–0.04	0.055
**Random effects**					
	*σ* ^2^	0.03			
	*τ*_00 Stimulus_	0.01			
	*τ*_00 Participant_	0.01			
	N _Participant_	58			
	N _Stimulus_	116			
	Observations	5,369			
	Marginal R^2^/Conditional R^2^	0.188/0.505			

Significant fixed effects are labeled for ease of reference within the text.

#### Comparison of Individual Differences Model With Group Model

The model examining individual differences (IDs) among bilingual participants’ RTs (Table 8) is qualitatively similar to the model comparing across all three participant groups (Table 4). Setting aside for a moment effects of the language experience predictors, the primary difference is that the IDs analysis returned an interaction between Noise Condition and Group such that the overall effect of noise (6) was greater for the Heritage as compared to the L2 group, and the effects of energetic vs. informational masking were reversed (7), such that the Heritage group suffered more in two-talker babble, while the L2 group suffered more in speech-shaped noise. This interaction is likely significant in the IDs model due to its less complex random effects structure; the group model contains random by-participant and by-stimulus slopes for the effect of Noise Condition, while the IDs model does not.

**Figure 4:**
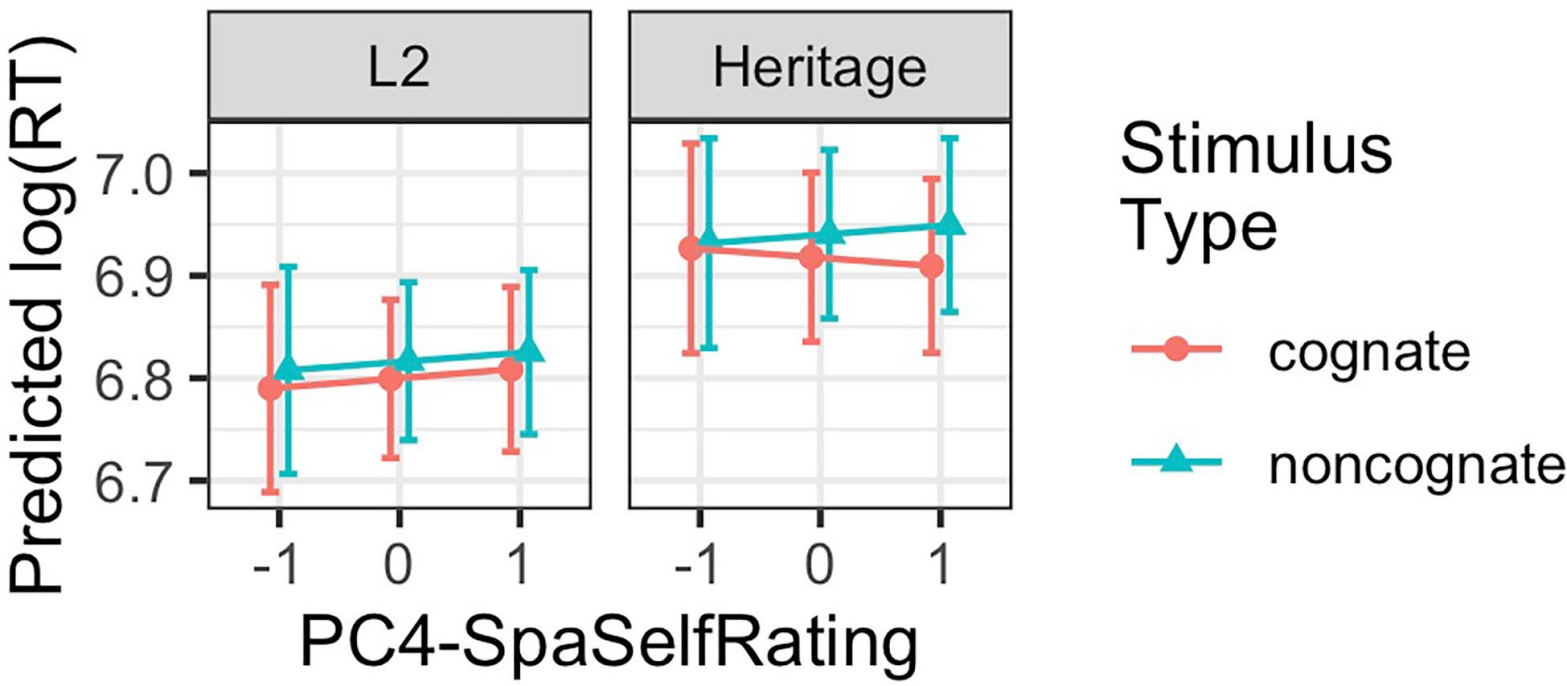
Predicted response times (estimated marginal means with prediction intervals) showing the interaction of stimulus type, participant group, and PC4-SpanishSelfRating (centered and scaled), using the fitted model from Table 8.

**Figure 5:**
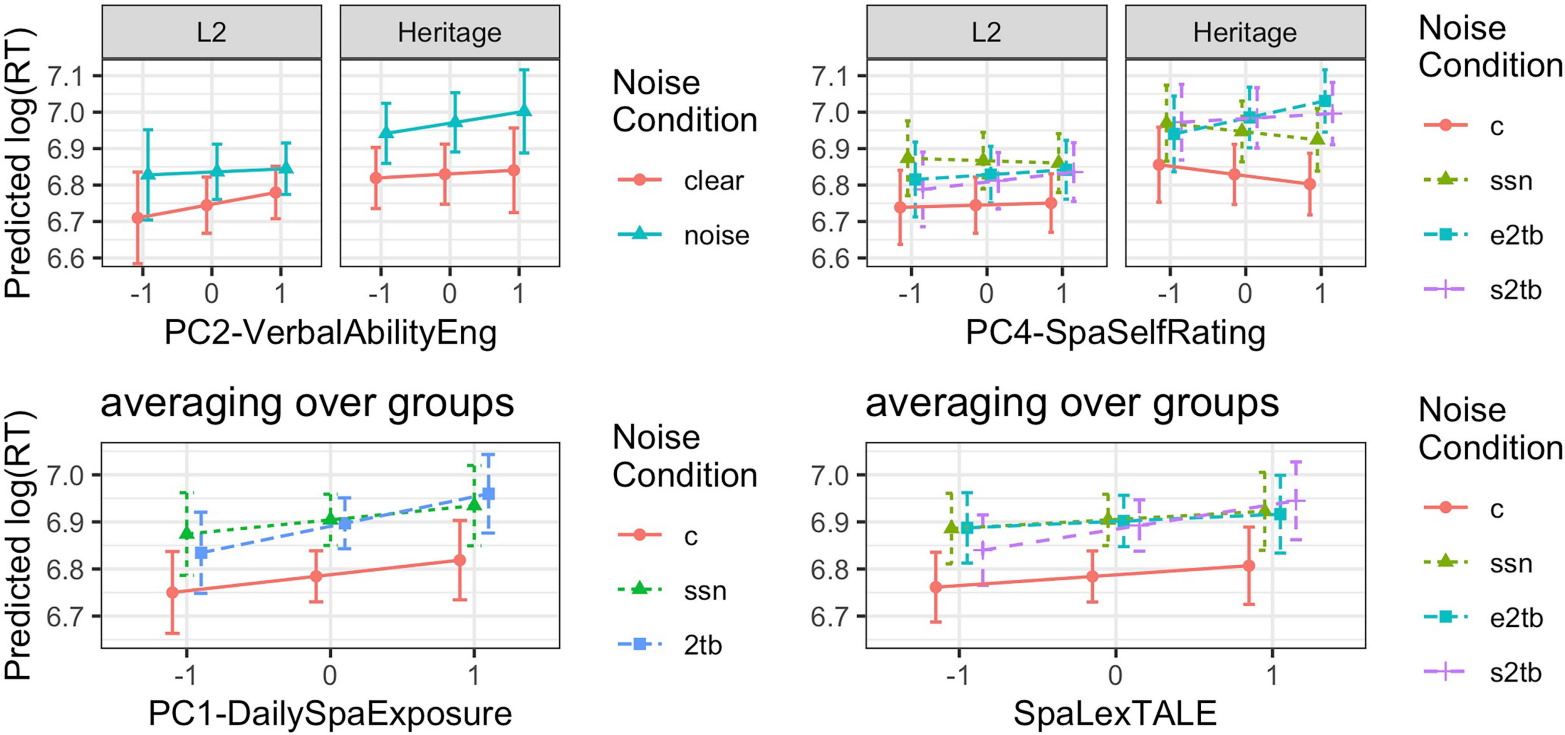
Predicted response times (estimated marginal means with prediction intervals) depicting significant predictors for noise masking effects in the fitted model in Table 8. All predictors have been centered and scaled, with estimated marginal means generated at the mean ± 1 *SD*. (c = clear, ssn = speech-shaped noise, e2tb = English two-talker babble, and s2tb = Spanish two-talker babble).

Turning to the language experience predictors, the coefficients for NoiseC2*PC1 (8), NoiseC1*PC4 (9), NoiseC2*PC4 (10), NoiseC3*SpaLexTALE (11), NoiseC1*BilingGroup*PC2, (13), and all three noise comparisons for BilingGroup*PC4 (14–16) differed reliably from zero. The coefficients for CogStatus*PC4 (12) and BilingGroup*CogStatus*PC4 (17) differed marginally from zero. Figures 4, 5 visualize the prediction intervals involving these model coefficients.

#### Individual Differences in the Cognate Effect

Similar to the group analysis, there was no main effect of Cognate Status and no interaction between Cognate Status and Noise Condition. Figure 4 depicts the interaction of Cognate Status, PC4-SpaSelfRating, and Group. For L2 Spanish listeners, the difference in RTs between cognates and noncognates remained constant across values of PC4-SpaSelfRating and was not statistically significant (estimate = −0.02, *SE* = 0.02, *p* = 0.28). For Heritage listeners, the difference between cognates and noncognates was nonexistent at lower values of PC4-SpaSelfRating (for the lowest values, estimate = −0.01, *SE* = 0.02, *p* = 0.91; for average values, estimate = −0.02, *SE* = 0.02, *p* = 0.22), and significant at the highest values (estimate = −0.04, *SE* = 0.02, *p* = 0.03).

#### Individual Differences in Masking Effects

The lower left panel of Figure 5 depicts the interaction of Noise Condition and PC1-DailySpaExposure (8), averaging across listener groups and the two-talker babble conditions in line with the model. On average, RTs in the clear were slower for high vs. low values of PC1-DailySpaExposure, but this difference was not significant (estimate = 0.06, *SE* = 0.07, *p* = 0.35). The recognition deficit for the two-talker babble conditions (i.e., the difference in RTs between the 2 TB and clear conditions) was smaller at low values of PC1-DailySpaExposure as compared to high values (estimated difference = 0.06, *SE* = 0.02, *p* = 0.03), while the recognition deficit for speech-shaped noise remained constant across values of PC1-DailySpaExposure (estimated difference = −0.01, *SE* = 0.03, *p* = 0.95).

The lower right panel of Figure 5 depicts the two-way interaction of Noise Condition with SpaLexTALE (11). Across listener groups, the recognition deficit in S2TB increased along with SpaLexTALE scores (estimate = 0.06, *SE* = 0.02, *p* = 0.03), while recognition deficits remained constant for both the SSN (estimate = −0.01, *SE* = 0.02, *p* = 0.97) and E2TB conditions (estimate = −0.02, *SE* = 0.02, *p* = 0.83).

The upper left panel of Figure 5 depicts the three-way interaction of Group and Noise Condition with PC2-VerbalAbilityEng (14), with the three noise conditions averaged together in line with the statistical model; PC2-VerbalAbilityEng did not interact with masker type. For L2 listeners, RTs in the clear tended to increase along with PC2-VerbalAbilityEng, while for Heritage listeners, RTs in noise tended to increase along with PC2-VerbalAbilityEng, though neither of these trends were significant (for L2, estimate = 0.07, *SE* = 0.07, *p* = 0.51; for Heritage, estimate = 0.06, *SE* = 0.06, *p* = 0.50). However, L2 listeners showed comparatively smaller noise deficits at high (vs. low) values of PC2 (estimate = −0.05, *SE* = 0.02, *p* = 0.02), while Heritage listeners trended toward the opposite pattern (estimate = 0.04, *SE* = 0.02, *p* = 0.07).

The upper right panel of Figure 5 depicts the three-way interaction of Group, Noise Condition, and PC4-SpaSelfRating (14–16). For L2 listeners, the recognition deficits in all three types of noise remained constant across values of PC4-SpaSelfRating (for SSN, estimate = −0.03, *SE* = 0.02, *p* = 0.45; for E2TB, estimate = 0.01, *SE* = 0.02, *p* = 0.81; and for S2TB, estimate = 0.04, *SE* = 0.02, *p* = 0.18). For Heritage listeners, recognition deficits increased along with PC4-SpaSelfRating in E2TB (estimate = 0.14, *SE* = 0.02, *p* < 0.001) and S2TB (estimate = 0.08, *SE* = 0.02, *p* < 0.001), while the deficit in SSN remained constant (estimate = 0.01, *SE* = 0.02, *p* = 0.97). Moreover, the difference in the recognition deficit for high vs. low levels of PC4-SpaSelfRating was greater for E2TB as compared to S2TB (estimate = 0.07, *SE* = 0.02, *p* = 0.001).

#### Interim Discussion for Individual Differences Analyses

We explored the effects of bilingual language experience on English word recognition in noise using principal component analysis. Three PCs (PC1-DailySpaExposure, PC2-VerbalAbilityEng, and PC4-SpaSelfRating) and the Spanish LexTALE scores improved the model’s ability to predict the masking effects, and PC4-SpaSelfRating also improved the model’s ability to predict the cognate effect.

For both bilingual listener groups, PC1-DailySpaExposure predicted the degree of informational masking, with lower PC1-DailySpaExposure values (i.e., greater daily Spanish exposure) associated with less masking from two-talker babble as compared to higher values of PC1-DailySpaExposure. Given that all participants were English-dominant, this result suggests that regular exposure to a non-dominant language may confer benefits in terms of coping with informational masking; we return to this idea in the General Discussion. The modulation in masking effects associated with Spanish LexTALE was also consistent across listener groups: bilinguals with higher Spanish LexTALE scores experienced more disruption from Spanish two-talker babble as compared to bilinguals with lower scores.

The effects of PC2-VerbalAbilityEng differed by listener group. For L2 listeners, lower values of PC2-VerbalAbilityEng (associated with a larger English vocabulary size) were associated with faster English word recognition in the clear, while for heritage listeners, lower values of PC2-VerbalAbilityEng (i.e., larger English vocabulary size) were associated with marginally reduced word recognition difficulties in noise. The fact that the effects of PC2-VerbalAbilityEng differed across groups indicates that English LexTALE scores index something different in L1 English-L2 Spanish listeners vs. heritage listeners, a point we return to in “Implications for Measures of Language Experience.”

PC4 corresponded most closely to Spanish self-ratings, with higher PC4 values corresponding to higher self-ratings. For L2 Spanish listeners, recognition deficits in noise were not modulated by PC4-SpaSelfRating. For heritage listeners, the deficits incurred in two-talker babble were greater for listeners with higher values of PC4-SpaSelfRating, and this effect was larger for English two-talker babble as compared to Spanish two-talker babble. This finding suggests that for heritage listeners, Spanish self-ratings may have actually been a better index of past English experience; we discuss this possibility in “Language Experience and Masking Effects.”

Finally, model comparisons returned a three-way interaction of PC4-SpaSelfRating, Participant Group, and Cognate Status. Pairwise comparisons of the estimated marginal means indicated that for L2 listeners, cognate effects did not differ reliably from zero, irrespective of PC4-SpaSelfRating. For heritage listeners, cognate effects differed reliably from zero only for those with the highest values of PC4-SpaSelfRating. Importantly, however, there was still no indication that cognate facilitation effects were modulated by the presence of background noise.

## General Discussion

### No Evidence for Increased Cross-Language Activation in Noise

In “Mechanisms by Which Cross-Language Activation Could Increase in Noise,” we hypothesized that one possible mechanism for increased cross-language activation in noise could be phonetically driven changes in the competitor activation process. Because competing noise makes the target phonetic input less recoverable (e.g., Bradlow and Alexander, 2007) and less reliable (e.g., McQueen and Huettig, 2012), non-target language competitors could be activated more strongly during the recognition process in noise than in the clear, leading to a larger cognate effect in noise.

Contrary to this proposal, cognate facilitation in this study did not increase in noise. This finding is striking in that it seems to run counter to assumptions made in the literature (e.g., Rogers et al., 2006). However, because this study examined performance in the more dominant language only, further studies should investigate whether cross-language activation increases in noise during word recognition in the less dominant language. Given the lack of support for the phonetically driven hypothesis, the present results suggest that if cross-language activation processes are altered by noise in the non-dominant language, factors involving executive function, language control, and the availability of cognitive resources will likely be responsible.

### Relating Language Experience to Auditory Word Recognition in Noise

#### Language Experience and Cognate Facilitation

While background noise did not impact cross-language activation, language experience did play a role. The RT analysis across all three listener groups showed that relative to the monolingual control group, bilinguals experienced cognate facilitation during English auditory word recognition. The individual differences analyses indicated that cognate facilitation was weak-to-nonexistent for L2 Spanish listeners and strongest for heritage listeners with the highest Spanish self-ratings. The two analyses may have differed in their estimation of the cognate effect for several reasons. The group analysis included a more complex random effect structure that was better able to account for stimulus duration and random by-participant differences, and the monolingual control comparison also helped account for sources of variation not attributable to bilingualism.

Interestingly, the magnitude of the cognate effect for heritage bilinguals was associated primarily with differences in Spanish self-ratings, and not with Spanish LexTALE scores. Since Spanish LexTALE scores predicted the degree of masking from Spanish two-talker babble, LexTALE seems to have served as a reasonable approximation of Spanish proficiency. One interpretation of the cognate finding is therefore that heritage bilinguals’ Spanish self-ratings reflected some latent aspect(s) of English exposure, an interpretation supported by the fact that English LexTALE scores also loaded onto PC4-SpaSelfRating. If heritage listeners with the highest Spanish self-ratings tended to have the weakest English lexical-phonetic representations, then we might predict them to show (1) slower RTs for noncognates, which do not benefit from cross-language activation and/or (2) faster RTs for cognates, which may be more strongly influenced by cross-language activation for listeners with the weakest representations. While there were weak trends in these directions, the cognate analysis itself does not enable us to say more on this point. However, the analysis of masking effects is relevant here, and we return to this issue below.

#### Language Experience and Masking Effects

The present study is one of relatively few to directly compare the effects of different noise types (see Scharenborg and van Os, 2019, for a recent review). Contrary to some findings (e.g., Cooke et al., 2010; Kilman et al., 2014), the group analysis here indicated that for most listeners, competing speech was no more disruptive than stationary noise, although the individual differences analyses painted a more nuanced picture. Importantly, only the group analysis incorporated random by-participant slopes for the effect of noise type. Such a model is likely to attribute differential effects of noise type to random variation in the listener population, when in fact they are partially explicable by systematic differences in language experience. Indeed, the individual differences analysis showed that differences in daily language exposure, Spanish proficiency, and English verbal ability all helped predict inter-individual differences in masking effects.

For both bilingual groups, greater daily Spanish exposure (corresponding to lower values of PC1-DailySpaExposure; see Figure 3) was associated with an improved ability to cope with informational masking. Interestingly, this finding identifies the quantity of non-dominant language exposure as the operative factor, and *not* proficiency in either the target or masking languages. This finding is compatible with the proposal that experience regulating the more dominant language, i.e., more time spent listening in the non-dominant language, hones the deployment of cognitive resources (e.g., Alladi et al., 2013). It also supports the argument that research examining relationships among bilingual language experience and cognitive function should move beyond static (i.e., proficiency-oriented) measures to focus more on dynamic measures of language experience (e.g., Beatty-Martínez et al., 2020). The question of *which aspects* of daily language experience may promote an improved ability to cope with informational masking should therefore be a topic of future research.

That greater daily Spanish exposure was associated with a reduced noise deficit seems to run counter to von Hapsburg and Bahng (2009), who found that L2 immersion was associated with more impaired L1 word recognition in noise. Several differences between studies should be noted here. First, the current listeners were not in any sense fully immersed in the non-dominant language. The proportion of daily Spanish exposure averaged 0.19 and ranged from 0.01 to 0.57; contextual support for the dominant language was thus quite high. Second, the modulation of the noise deficit found here was restricted to the two-talker babble conditions, indicating a relationship specifically between non-dominant language exposure and the ability to cope with informational masking; von Hapsburg and Bahng examined only energetic masking. Future research should further explore the relationships among non-dominant language exposure, informational masking, and domain-general executive function.

In addition to the effect of daily Spanish exposure on informational masking, Spanish proficiency (i.e., Spanish LexTALE scores) predicted the degree of informational masking incurred by Spanish babble, an effect that was equivalent across the two bilingual groups. This finding is roughly in line with previous SPIN findings (Imai et al., 2005; Van Engen, 2010; Brouwer et al., 2012; Kilman et al., 2014), but it extends these in several ways; namely, by moving beyond group-level analyses to relate the objectively measured vocabulary knowledge of individual listeners to the magnitude of the interference effect, and also by identifying such an effect during word recognition in the dominant language.

With respect to effects of English language experience, heritage listeners’ overall noise deficits were marginally related to PC2-VerbalAbilityEng, which was primarily composed of English LexTALE scores. Informational masking was also greater overall for heritage listeners as compared to the L2 Spanish group, and it increased along with values of PC4-SpaSelfRating. As alluded to previously, the fact that PC4-SpaSelfRating predicted informational masking independently of Spanish LexTALE scores suggests that PC4-SpaSelfRating reflected some aspect of English experience. Importantly, increasing values of PC4-SpaSelfRating were associated with more sharply increasing disruption from competing English speech as compared to competing Spanish speech (Figure 5, upper right); this suggests that the relative weakness of the target English representations may be most relevant here, and not the strength of competing Spanish representations. Under this interpretation, listeners with the weakest English representations were the most susceptible to informational masking, and informational masking was strongest when the target and competing speech were most similar (Van Engen, 2010). The same listeners were also most likely to show cognate facilitation effects. Taken together, the results suggest that (1) for heritage listeners, PC4-SpaSelfRating likely indexed some aspect of early-acquired English lexical-phonetic knowledge and (2) this knowledge was distinct from daily language exposure (i.e., PC1-DailySpaExposure), Spanish age of acquisition (PC3-SpaAoA), or Spanish vocabulary knowledge (SpaLexTALE). This in turn suggests that cognate facilitation for heritage listeners was likewise driven by differences in early-acquired English lexical-phonetic knowledge that were not captured by these other metrics.

Finally, returning to the lack of differentiation among noise conditions in the group analysis, differences in task difficulty across studies may have also played a role. Calandruccio et al. (2010) argued that effects of informational masking were greatest when the speech comprehension system was most stressed, i.e., when task difficulty was highest. Previous studies employed tasks that may have been more cognitively taxing than the present experiment; Cooke et al. (2010) used a 24AFC consonant identification task, and Kilman et al. (2014) used a sentence repetition paradigm. The group analysis may therefore have found no differences across noise types because lexical decision in the native language does not generally incur enough cognitive load to reveal significant effects of informational masking. The findings from the individual differences analysis involving heritage listeners support this interpretation; the combination of relatively weaker English lexical-phonetic representations and relatively stronger competition from Spanish representations is likely to have increased task difficulty for some heritage listeners, making them particularly susceptible to informational masking.

### Implications for Measures of Language Experience

Principal component analysis suggested four main axes along which the English-dominant bilinguals in this study varied: daily exposure to Spanish vs. English (PC1-DailySpaExposure; 43% of the total variance in language experience measures); verbal ability primarily in the more dominant language, English (PC2-VerbalAbilityEng; 19% of variance); Spanish age of acquisition (PC3-SpaAoA; 15% of variance); and self-rated proficiency in the non-dominant language, Spanish (PC4-SpaSelfRating; 10% of variance). Three of these helped explain variance in English word recognition in noise; age of acquisition, notably, did not. That measures capturing more nuanced aspects of accumulated experience were predictive, and not AoA *per se*, is broadly consistent with perspectives emphasizing the role of plasticity and continued learning over the life span (Baum and Titone, 2014; Flege and Bohn, 2021).

The fact that Spanish LexTALE was not uniquely associated with any single PC, but rather loaded into multiple components, indicates that receptive vocabulary knowledge in the non-dominant language was not a unique axis of variation for the bilinguals in this study. However, Spanish LexTALE scores predicted the degree of masking incurred by competing Spanish speech. Given previous findings that LexTALE performance correlates with other aspects of proficiency in the non-dominant language (Lemhöfer and Broersma, 2012), Spanish LexTALE likely predicted Spanish masking because it indexed listeners’ facility in deriving meaning from competing Spanish speech.

However, the fact that PC2-VerbalAbilityEng (composed predominantly of English LexTALE scores) yielded different effects for L2 vs. heritage listeners indicates that LexTALE scores may only provide a useful measure of dominant-language linguistic knowledge for certain populations of bilinguals. If L2 listeners’ English language knowledge was more likely to be at ceiling, then performance in the two lexical decision tasks (LexTALE and SPIN) may have reflected individual differences in processing speed, attentional focus, or other domain-general attributes. On the other hand, heritage listeners’ performance in the two lexical decision tasks may have tended to reflect variation in English lexical knowledge. If correct, this adds nuance to Ferré and Brysbaert's (2017) suggestion that LexTALE can discriminate among bilinguals at the high end of the proficiency range even for the more dominant language; LexTALE may specifically provide an appropriate proficiency measure only for bilinguals whose dominance has shifted over time, or perhaps for bilinguals whose language input has been more equally shared across languages over the life span.

The effects of PC4-SpaSelfRating also differed across groups, aligning with recent demonstrations that self-ratings can reflect different aspects of experience for different populations (Tomoschuk et al., 2019). The results therefore support calls to incorporate objective language proficiency measures (Kilman et al., 2014; Warzybok et al., 2015; Scharenborg et al., 2018), with the caveats that (1) caution is warranted in using LexTALE as a measure of linguistic knowledge in the more dominant language and (2) self-reported measures still have an important role to play (see Gollan et al., 2011b and Gullifer et al., 2021, for more nuanced discussion).

While the present work has begun to identify how different aspects of linguistic experience impact bilingual SPIN, a more complete understanding will require identifying how experience impacts specific components of the recognition process (see Beatty-Martínez and Titone, 2021 and Green and Wei, 2014 for similar arguments regarding executive function). Work by Krizman et al. (2017) has begun separating out the relevant components of auditory-linguistic processing. Future work should ideally merge these streams of inquiry.

### Implications for Heritage Speaker Populations

The present results add nuance to previous findings regarding speech perception in heritage speaker populations. On average, heritage listeners were no slower than monolinguals to recognize words in English, though they were more affected by the presence of noise and by informational masking in particular as compared to L1 English-L2 Spanish listeners. The results therefore align with previous literature in suggesting that heritage listeners’ lexical-phonetic knowledge in the more dominant language may in some cases be less robust as compared to listeners who acquired a single language in childhood (Mayo et al., 1997; Rogers et al., 2006; Morini and Newman, 2020). However, they also demonstrate that in some cases, such effects may be small-to-negligible. Future studies should therefore include large sample sizes to guard against inappropriately concluding that heritage listeners are uniformly disadvantaged in word recognition. They should also combine careful and detailed measures of language experience in order to further disentangle the factors influencing language processing behavior in this population.

## Conclusion

In addition to being one of relatively few studies to report cognate activation during auditory word recognition (Woutersen et al., 1995; Blumenfeld and Marian, 2007), this study showed that cross-language activation processes were not affected by background noise during word recognition in the dominant language. A detailed exploration of individual differences indicated that the ability to cope with informational masking was particularly subject to modulation by language experience. Taken together, the findings confirm the highly interactive nature of bilingual language processing and suggest that auditory word recognition processes in the native language remain susceptible to influence from linguistic experience throughout the lifespan.

## Data Availability Statement

The raw data supporting the conclusions of this article will be made available by the authors, without undue reservation.

## Ethics Statement

This study involving human participants was reviewed and approved by the Human Research Protection Office, University of Pittsburgh. The participants provided their written informed consent to participate in this study.

## Author Contributions

All aspects of the study were carried out by MF (study design, stimulus preparation, data collection, analysis, and manuscript preparation) with help from research assistants as noted in the Acknowledgments.

## Funding

This research was supported by a Language Learning Early Career Grant to MF.

## Conflict of Interest

The author declares that the research was conducted in the absence of any commercial or financial relationships that could be construed as a potential conflict of interest.

## Publisher’s Note

All claims expressed in this article are solely those of the authors and do not necessarily represent those of their affiliated organizations, or those of the publisher, the editors and the reviewers. Any product that may be evaluated in this article, or claim that may be made by its manufacturer, is not guaranteed or endorsed by the publisher.

## Footnotes

^1^ www.prolific.co

^2^ www.gorilla.sc
